# Microwave-assisted efficient and facile synthesis of tetramic acid derivatives via a one-pot post-Ugi cascade reaction

**DOI:** 10.3762/bjoc.16.63

**Published:** 2020-04-09

**Authors:** Yong Li, Zheng Huang, Jia Xu, Yong Ding, Dian-Yong Tang, Jie Lei, Hong-yu Li, Zhong-Zhu Chen, Zhi-Gang Xu

**Affiliations:** 1 College of Pharmacy, National & Local Joint Engineering Research Center of Targeted and Innovative Therapeutics, Chongqing Key Laboratory of Kinase Modulators as Innovative Medicine, Chongqing University of Arts and Sciences, Chongqing 402160, China; 2 Department of Pharmaceutical Sciences, College of Pharmacy, University of Arkansas for Medical Sciences, Little Rock, AR 72205, USA

**Keywords:** Dieckmann cyclization, multicomponent reactions, nitrogen heterocycles, one-pot reaction, Ugi reaction

## Abstract

A facile microwave-assisted method for the synthesis of tetramic acid derivatives has been developed through an Ugi/Dieckmann cyclization strategy with DBU. This two-step one-pot procedure afforded the targeted tetramic acid analogues in good yields. With commercially available Ugi starting materials, microwave irradiation, a simple operation, excellent yields, and a broad scope, this reaction has the potential to produce a large number of tetramic acid analogues, which cannot be easily accessed by the classic synthetic methods.

## Introduction

**Figure 1:**
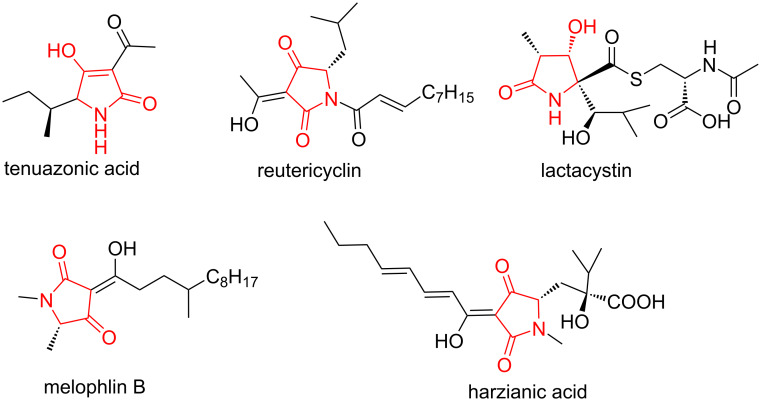
Structures of natural tetramic acid derivatives with more clinical relevance.

Nitrogen-containing heterocycles are privileged scaffolds that can be found in a wide variety of bioactive pharmaceuticals and natural products [1]. For this reason, research towards the development of novel and efficient strategies for the construction of these compounds represents one of the most active areas in synthetic organic chemistry [2–4]. Notably, one of these nitrogen-containing heterocycles is tetramic acid which contains a pyrrolidine-2,4-dione moiety. Many natural products have this key structure moiety (Figure 1) [5–7]. Because of their active cyclic keto-enol structure, the heterocyclic tetramic acid compounds exhibit a wide range of biological activities, including antibiotic [8], antiviral [9], antifungal [10], phytotoxic [11], cytotoxic [12–13], and enzyme inhibitory activities against bacterial DNA-directed RNA polymerase [14]. The wide range of biological activity and structural variation of this class of compounds makes them attractive for library generations and sequential biological testing [15–16]. Although several classical synthetic procedures were developed [17–21], more effective and facile syntheses for a large number of compounds are still required for high throughput screening in medicinal chemistry.

Multicomponent reactions (MCRs) [22–24] are emerging tools for assembling complex molecules in a rapid and efficient manner. The four-component Ugi reaction followed by a suitable post modification of [25–26] has become a popular research field for diversity-oriented synthesis [27–29] of complex heterocyclic compounds. Recent advance has been focused in the area toward the development of one-pot [30–32] Ugi cascade protocol [33–34] as the powerful synthetic strategy for the construction of increasingly complex heterocyclic compounds in the absence of metal catalysts [35–37]. These new processes improved the overall efficiency with shorter reaction times and lower costs of synthetic preparations, and minimized the amount of waste generated.

In recent years, we used the Ugi four-component reaction as a main tool to generate nitrogen-containing heterocycles [38–39]. Spatz’ team reported a novel two-step synthetic procedure for the preparation of substituted tetramic acid derivatives via an Ugi/Dieckmann reaction [40]. A leaving amide that was derived from the isonitrile, provided the tetramic acid core under strong inorganic basic conditions. In this method, a convertible isonitrile promoted the cyclization, but restricted the scope of structural diversity. For this reason, it is desirable to replace the amide with a better leaving group, an ester group, which will undergo the Dieckmann reaction more effectively to form a five-membered ring. It is worth to mention that our hypothesis is complementary to Spatz’ work. To test our hypothesis, glyoxylate ethyl ester and phenyl acetic acid were selected as model substrates to evaluate the feasibility of this cyclization. Herein, we report the development of an efficient method for the synthesis of novel tetramic acid derivatives with potential interesting biological activities via an Ugi/Dieckmann cyclization strategy.

## Results and Discussion

**Scheme 1:**
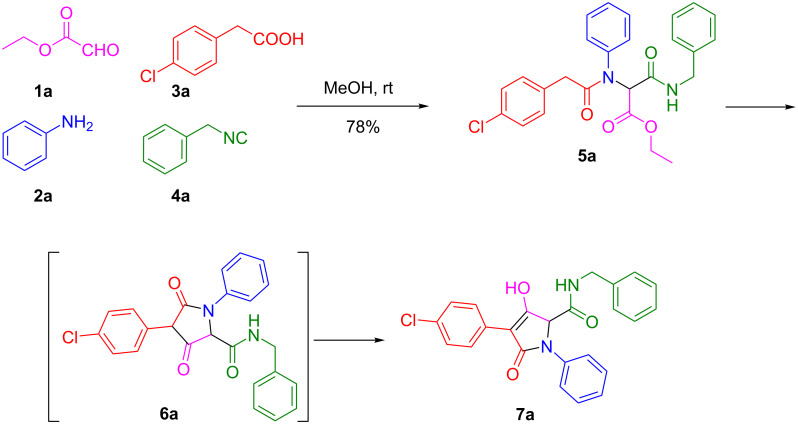
Synthetic strategy of compound **7a**.

We initially stirred a mixture of ethyl glyoxylate (**1a**), aniline (**2a**), 2-(4-chlorophenyl)acetic acid (**3a**), and benzylisonitrile (**4a**) in methanol at room temperature overnight (Scheme 1). The reaction provided the crude Ugi adduct **5a** after the removal of methanol under reduced pressure. The crude residue was then exposed to a series of different cyclization reaction conditions. First, based on the previous works on the Dieckmann cyclization reaction [40–41], we investigated the effect of different inorganic and organic bases (Table 1). It was interesting to note that all of the bases tested in the study afforded the desired enol tetramic acid derivative **7a** after the reaction was heated under the microwave irradiation conditions. The ketone compound **6a** could be the intermediate which would be quickly converted into the more stable enol product **7a**.

**Table 1:** Optimization of the Dieckmann reaction for compound **7a**.

entry	solvent	base (2.0 equiv)	temp. (°C)	time	Yield (%)^a^

1	DMF^b^	Cs_2_CO_3_	MW 100	10 min	68
2	DMF	NaOH	MW 100	10 min	72
3	DMF	Na_2_CO_3_	MW 100	10 min	56
4	DMF	TEA	MW100	10 min	trace
5	DMF	TEA	MW 120	10 min	46
6	DMF	DBU	MW 100	10 min	70
7	DMF	DMAP	MW 100	10 min	trace
8	DMF	DMAP	MW 120	10 min	trace
9	toluene	DBU	MW 100	10 min	34
10	MeOH	DBU	MW 100	10 min	62
11	DMSO	DBU	MW 100	10 min	62
12	DCE	DBU	MW 100	10 min	26
13	DMF	DBU	MW 80	10 min	53
14	DMF	DBU	MW 120	10 min	85
15	DMF	DBU	MW 140	10 min	72
16	DMF	DBU	120	10 min	trace

^a^Yield of the isolated product. ^b^MW = microwave irradiation, DMF = *N*,*N*-dimethylformamide, DBU = 1,8-diazabicyclo[5.4.0]undec-7-ene, TEA = trimethylamine, DMAP = 4-dimethylaminopyridine.

When the inorganic bases, such as Cs_2_CO_3_, NaOH, and Na_2_CO_3_, were tested, the desired product **7a** was obtained in 56–72% yields (Table 1, entries 1–3). In contrast, the organic bases TEA and DMAP (Table 1, entries 4, 5, 7, 8) failed to afford comparable or better yields. However, when DBU, an organic base, was used, the yield of **7a** was significantly increased (Table 1, entry 6). We then investigated the effect of solvent and temperature for the reaction. The results tested in different solvent systems were summarized in Table 1, entries 9–12. DMF was superior to other solvents with the highest yield. Then the microwave irradiation temperature was varied: at 80 °C for 10 min, a large fraction of the Ugi adduct **5a** was still present in the product mixture (LC–MS determination); at 120 °C, the desired compound **7a** was obtained in the highest yield of 85%. However, at 140 °C, the yield dropped to 72%, probably due to decomposition of the product. When we used an traditional oil bath to heat the mixture for 10 min, only a trace amount of compound **7a** was detected by LC–MS (Table 1, entry 16). Compared with Table 1, entry 14, the microwave irradiation reduced the reaction time and improved the reaction efficiency. Hence, optimized reaction conditions were obtained: the reaction was heated under microwave irradiation at 120 °C for 10 min in DMF (Table 1, entry 11).

**Scheme 2:**
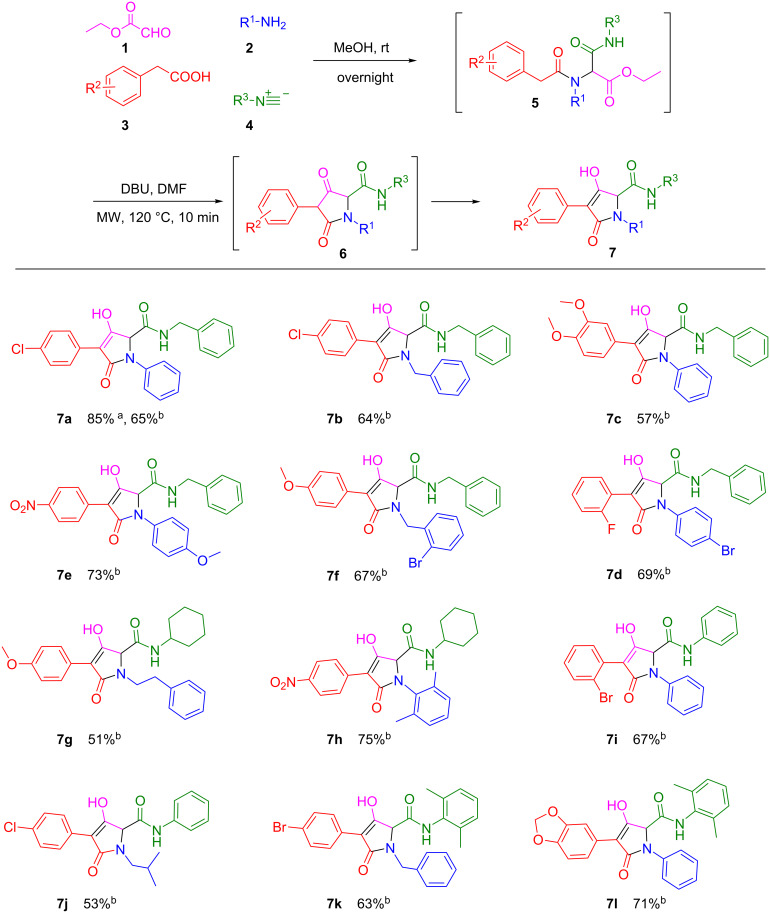
Scope of the Ugi/Dieckmann cyclization reaction route to lead to pyrrolopyridinones **7a**–**l**. ^a^Yield of the isolated product of the Dieckmann cyclization reaction. ^b^Overall yield over two steps of isolated product.

With the optimized reaction conditions in hand, we proceeded to investigate the scope and limitation of this reaction using a variety of different starting materials, which resulted in the preparation of a small collection of generic compounds **7a**–**l**. In all of the cases, the initial Ugi product **5** was used directly in the next step without purification after removing the solvent under a gentle stream of nitrogen. Different amines, acids, and isonitriles were subjected to the one-pot protocol to give the corresponding tetramic acid derivatives **7** from the ketone intermediates **6** in good yields over two steps (51–75%) after the purification of the products by column chromatography (Scheme 2). It was found that the reaction showed a good functional group tolerance; no matter if R^2^ was electron-withdrawing or electron-donating, the target compounds could all be obtained in good yields. And aromatic or aliphatic amines (as R^1^) all gave the targeted products with good yields. As such, this newly developed protocol provided a facile access to tetramic acid derivatives in one pot, and it could be used for the preparation of chemical compound libraries containing a large number of tetramic acid analogues for subsequent high throughput screenings.

**Scheme 3:**
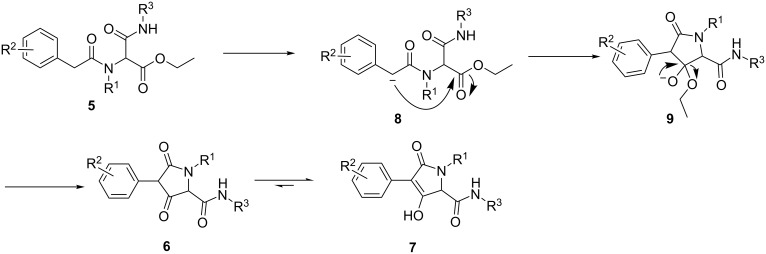
Postulated reaction mechanism.

Based on these results, a reaction mechanism was therefore postulated in Scheme 3. Deprotonation of the α-carbon next to the carbonyl group generates a carbanion **8**, which then undergoes a nucleophilic attack to the carbonyl carbon of the ester to give a cyclic enol **9**. Leaving of the ethoxy group forms the 2,4-dione compound **6**, which would be quickly converted into **7,** a more stable enol form due to its conjugation to the phenyl ring.

## Conclusion

In summary, we have developed a new method for the construction of tetramic acid derivatives by using a one-pot Ugi/Dieckmann cyclization protocol. In comparison with the existing protocols, our method is more straightforward and broadly applicable. With the mild reaction conditions, good yields, and commercial availability of the starting materials, we believe that this method represents a valuable tool for the synthesis of tetramic acid analogues and other heterocyclic compounds.

## Experimental

In a similar manner as described in [38] the reactions were conducted as follows: To a magnetically stirred solution of ethyl glyoxylate (1.0 mmol) in MeOH (1.0 mL) was added the amine (0.5 mmol) in a 5 mL microwave vial, and the resulting solution was stirred at room temperature for 10 min. The acid (0.50 mmol) and the isocyanide (0.50 mmol) were then added separately. The mixture was stirred at room temperature overnight, and the progress of the reaction was monitored by TLC. The solvent was removed under a stream of nitrogen, and the residue was dissolved in DMF (3.0 mL), and then DBU (1.0 mmol) was added. The mixture was then placed in a microwave synthesizer and heated to 120 °C for 10 min. The mixture was then cooled to room temperature, and the residue was dissolved in EtOAc (15.0 mL). The solution was washed with brine, and the organic layer was dried with MgSO_4_ and filtered. The filtrate was concentrated, which gave a residue that was purified by silica gel column chromatography (ethyl acetate/hexane, 10–60%) to afford the product **7a**–**l**.

## Supporting Information

File 1 General information, general experimental procedure, characterization data, and copies of ^1^H and ^13^C NMR spectra.
