# Supplementary material for: Microwave-assisted efficient and facile synthesis of tetramic acid derivatives via a one-pot post-Ugi cascade reaction
Source: Beilstein J Org Chem. 2020 Apr 9;16:663–9. doi: 10.3762/bjoc.16.63 (PMC7155898; doi:10.3762/bjoc.16.63)
Supplement: File 1 — General information, general experimental procedure, characterization data, and copies of 1H and 13C NMR spectra. [file Beilstein_J_Org_Chem-16-663-s001.pdf]

BEILSTEIN JOURNAL OF ORGANIC CHEMISTRY

Supporting Information

for

Microwave-assisted efficient and facile synthesis of tetramic acid derivatives via a one-pot post-Ugi cascade reaction

Yong Li, Zheng Huang, Jia Xu, Yong Ding, Dian-Yong Tang, Jie Lei, Hong-yu Li, Zhong-Zhu Chen and Zhi-Gang Xu

Beilstein J. Org. Chem. **2020,** *16,* 663–669. doi:10.3762/bjoc.16.63

General information, general experimental procedure, characterization data, and copies of ^{1}H and ^{13}C NMR spectra

License and Terms: This is a supporting information file under the terms of the Creative Commons Attribution License (http://creativecommons.org/licenses/by/4.0). Please note that the reuse, redistribution and reproduction in particular requires that the authors and source are credited.
The license is subject to the *Beilstein Journal of Organic Chemistry* terms and conditions: (https://www.beilstein-journals.org/bjoc)

Table of contents

1.**General information** S3

2.**Experimental procedures and characterization data**..............................S4

3.References S9

4.**Copies of ^{1}H and ^{13}C NMR spectra** S10

General information

^{1}H and ^{13}C NMR were recorded on a Bruker 400 spectrometer. ^{1}H NMR data are reported as follows: chemical shift in ppm (δ), multiplicity (s = singlet, d = doublet, t = triplet, m = multiplet), coupling constant (Hz), relative intensity. ^{13}C NMR data are reported as follows: chemical shift in ppm (δ). LC/MS analyses were performed on a Shimadzu-2020 LC-MS instrument using the following conditions: Shim-pack VP-ODS C18 column (reversed phase, 150 × 4.6 mm); a linear gradient from 10% water and 90% acetonitrile to 75% acetonitrile and 25% water over 6.0 min; flow rate of 0.5 mL/min; UV photodiode array detection from 200 to 400 nm. High-resolution mass spectra (HRMS) were recorded on a Thermo Scientific Q Exactive Plus System. The products were purified by Biotage Isolera™ Spektra Systems and hexane/EtOAc solvent systems. All reagents and solvents were obtained from commercial sources and were used without further purification. All microwave irradiation experiments were carried out in a Biotage® Initiator Classic microwave apparatus with continuous irradiation power from 0 to 400 W with utilization of the standard absorbance level of 250 W maximum power. The reactions were carried out in 10 mL glass tubes, sealed with microwave cavity. The reaction was irradiated at a required ceiling temperature using maximum power for the stipulated time. Then, it was cooled to 50 °C with gas jet cooling.

All reagents, unless otherwise stated, were used as received from commercial suppliers.

Experimental procedures and characterization data

General procedure for tetramic acid derivatives 7a–l: In a similar manner as described in reference [1] the reactions were conducted as follows: To a magnetically stirred solution of the ethyl glyoxylate (1.0 mmol) in MeOH (1.0 mL) was added the amine (0.5 mmol) in a 5 mL microwave vial, and the resulting solution was stirred at room temperature for 10 min. The acid (0.50 mmol) and the isocyanide (0.50 mmol) were then added separately. The mixture was stirred at room temperature overnight, and the progress of the reaction was monitored by TLC. The solvent was removed under a stream of nitrogen, and the residue was dissolved in DMF (3.0 mL), and then DBU (1.0 mmol) was added. The mixture was then placed in a microwave synthesizer and heated to 120 °C for 10 min. The mixture was then cooled to room temperature, and the residue was dissolved in EtOAc (15.0 mL). The solution was washed with brine, and the organic layer was dried with $MgSO_4$ and filtered. The filtrate was concentrated, which gave a residue that was purified by silica gel column chromatography (ethyl acetate/hexane, 10–60%) to afford the product **7a**–**l**.

***N*-Benzyl-4-(4-chlorophenyl)-3-hydroxy-5-oxo-1-phenyl-2,5-dihydro-1*H*-pyrrole-2-carboxamide (7a):** Yellow solid (65% yield).

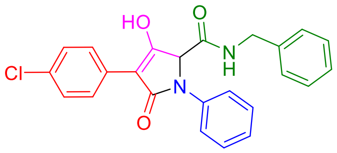

^{1}H NMR (400 MHz, DMSO-d_6) δ 12.23 (s, 1H, OH), 8.86 (t, J = 5.8 Hz, 1H, NH), 7.93 (d, J = 8.5 Hz, 2H, ArH), 7.63 (d, J = 8.0 Hz, 2H, ArH), 7.45 (d, J = 8.5 Hz, 2H, ArH), 7.35 (t, J = 7.8 Hz, 2H, ArH), 7.23 – 7.16 (m, 3H, ArH), 7.12 (t, J = 7.3 Hz, 1H, ArH), 7.05 – 6.96 (m, 2H, ArH), 5.37 (s, 1H，CH), 4.31 – 4.23 (m, 2H，CH_2). ^{13}C NMR (100 MHz, DMSO-d_6) δ 170.33, 166.73, 165.99, 139.03, 138.59, 131.07, 130.73, 129.69, 129.12, 128.59, 128.35, 127.25, 124.02, 120.93, 104.39, 64.55, 55.37, 42.94. HRMS: m/z calculated for $C_{24}H_{20}ClN_2O_3$ $[M+H]^+$: 419.11570, found 419.11606. The analytical data matched those reported in the literature [2].

N,1-Dibenzyl-4-(4-chlorophenyl)-3-hydroxy-5-oxo-2,5-dihydro-1H-pyrrole-2-carboxamide (7b):

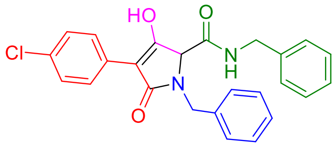

Yellow solid (64% yield).^{1}H NMR (400 MHz, DMSO-d_6) δ 11.98 (s, 1H, OH), 8.77 (s, 1H, NH), 7.99 (d, J = 8.7 Hz, 2H, ArH), 7.45 – 7.40 (m, 2H, ArH), 7.33 (t, J = 7.3 Hz, 4H, ArH), 7.29 – 7.22 (m, 4H, ArH), 7.17 (d, J = 7.1 Hz, 2H, ArH), 4.93 (d, J = 15.7 Hz, 1H, CH_2), 4.49 (s, 1H, CH), 4.30 –4.22 (m, 2H, CH_2), 4.00 (d, J = 15.7 Hz, 1H, CH_2). ^{13}C NMR (100 MHz, DMSO-d_6) δ 171.61, 166.42, 165.89, 139.16, 138.07, 131.10, 130.86, 129.28, 128.96, 128.76, 128.31, 127.91, 127.85 127.58, 127.42 103.69, 63.74, 44.18, 43.22. HRMS: m/z calculated for $C_{25}H_{22}ClN_2O_3$ [M+H]$^{+}$: 433.13135, found 433.13159. The analytical data matched those reported in the literature [2].

N-Benzyl-4-(3,4-dimethoxyphenyl)-3-hydroxy-5-oxo-1-phenyl-2,5-dihydro-1 H-pyrrole-2-carboxamide(7c):

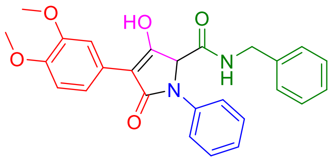

Yellow solid (57% yield). ^{1}H NMR (400 MHz, DMSO-d_6) δ 11.83 (s, 1H, OH), 8.86 (t, J = 5.8 Hz, 1H, NH), 7.63 (d, J = 8.0 Hz, 2H, ArH), 7.49 (s, 1H, ArH), 7.40 (d, J = 8.3 Hz, 1H, ArH), 7.33 (t, J = 7.9 Hz, 2H, ArH), 7.21 (d, J = 6.5 Hz, 3H, ArH), 7.10 (t, J = 7.3 Hz, 1H, ArH), 7.04 (d, J = 5.9 Hz, 2H, ArH), 6.98 (d, J = 8.5 Hz, 1H, ArH), 5.33 (s, 1H, CH), 4.32 – 4.24 (m, 2H, CH_2), 3.77 (s, 6H, OCH_3). ^{13}C NMR (100 MHz, DMSO-d_6) δ 169.63, 165.15, 163.67 147.45, 146.92, 138.01, 137.84, 128.02, 127.51, 126.18, 122.76, 120.06, 119.58, 111.19, 110.91, 104.67, 63.16, 54.84, 41.84. HRMS: m/z calculated for $C_{26}H_{25}N_2O_5$ [M+H]$^{+}$: 445.17580, found 445.17474. The analytical data matched those reported in the literature[2].

N-Benzyl-1-(4-bromophenyl)-4-(2-fluorophenyl)-3-hydroxy-5-oxo-2,5-dihydro-1H-pyrrole-2-carboxamide (7d): Yellow solid (69% yield).^{1}H NMR (400 MHz, DMSO-d_6) δ 12.13 (s, 1H, OH), 9.02 (s, 1H, NH), 7.58 (d, J = 9.0 Hz, 2H, ArH), 7.50 (d, J = 8.9 Hz, 2H, ArH), 7.46 – 7.32 (m, 1H, ArH), 7.40 – 7.33 (m, 1H, ArH), 7.28 – 7.18 (m, 5H, ArH), 7.14 – 7.06 (m, 2H, ArH), 5.37 (s, 1H, CH), 4.31 (d, J = 5.9 Hz, 2H, CH_2). ^{13}C NMR (100 MHz, DMSO-d_6) δ 170.38, 165.89, 161.77, 159.31, 139.16, 138.45, 132.43, 132.39, 131.91, 129.55, 128.60, 127.39, 127.25, 124.20, 124.17 121.68, 118.79, 115.93, 115.72, 115.26, 100.79, 64.50, 42.90. HRMS: m/z calculated for $C_{24}H_{19}BrFN_2O_3$ $[M+H]^+$: 481.05576, found 481.05576. The analytical data matched those reported in the literature[2].

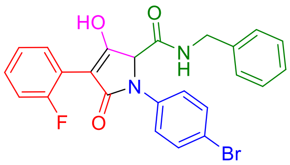

N-Benzyl-3-hydroxy-1-(4-methoxyphenyl)-4-(4-nitrophenyl)-5-oxo-2,5-dihydro-1H-pyrrole-2-carboxamide (7e): Yellow solid (73% yield).^{1}H NMR (400 MHz, DMSO-d_6) δ 8.80 (t, J = 5.9 Hz, 1H, NH), 8.36 (d, J = 9.1 Hz, 2H, ArH), 8.21 (d, J = 9.1 Hz, 2H, ArH), 7.48 (d, J = 9.0 Hz, 2H, ArH), 7.23 – 7.16 (m, 3H, ArH), 6.98 (d, J = 3.5 Hz, 2H, ArH), 6.92 (d, J = 9.0 Hz, 2H, ArH), 5.25 (s, 1H, CH), 4.36 – 4.18 (m, 2H, CH_2), 3.77 (s, 3H, OCH_3). ^{13}C NMR (100 MHz, DMSO-d_6) δ 169.35, 165.07, 155.27, 143.15, 139.74, 138.01, 130.50, 127.43, 126.22, 126.05, 125.77, 122.68, 122.61, 113.22, 64.78, 54.66, 41.76. HRMS: m/z calculated for $C_{25}H_{22}N_3O_6$ $[M+H]^+$: 460.15037, found 460.15076. The analytical data matched those reported in the literature[2].

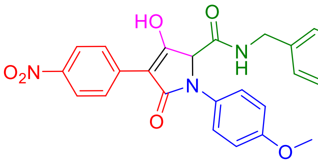

N-Benzyl-1-(2-bromobenzyl)-3-hydroxy-4-(4-methoxyphenyl)-5-oxo-2,5-dihydro-1H-pyrrole-2-carboxamide (7f): Yellow solid (67% yield).^{1}H NMR (400 MHz, DMSO-d_6) δ 11.54 (s, 1H, OH), 8.78 (s, 1H, NH), 7.87 (dd, J = 9.2, 2.4 Hz, 2H,

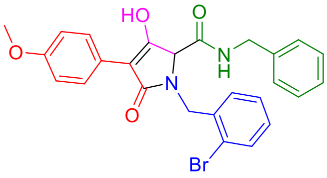

ArH), 7.63 (d, J = 7.9 Hz, 1H, ArH), 7.37 – 7.19 (m, 7H, ArH), 7.10 (d, J = 7.5 Hz, 1H, ArH), 6.94 (d, J = 7.7 Hz, 2H, ArH), 4.85 (d, J = 16.6 Hz, 1H, CH_2), 4.57 (d, J = 2.6 Hz, 1H,CH_2), 4.36 (dd, J = 14.7, 5.9 Hz, 1H), 4.20 (dd, J = 21.9, 11.3 Hz, 2H), 3.76 (s, 3H, OCH_3). ^{13}C NMR (100 MHz, DMSO-d_6) δ 172.21, 166.08, 164.52, 158.06, 139.08, 136.93, 132.98, 129.56, 129.21, 129.07, 128.75, 128.39, 127.82, 127.40, 124.43, 122.54, 113.78, 104.66, 63.83, 55.48, 44.78, 43.22. HRMS: m/z calculated for $C_{26}H_{24}BrN_2O_4$ $[M+H]^+$: 507.09140, found 507.09128.

***N*-Cyclohexyl-3-hydroxy-4-(4-methoxyphenyl)-5-oxo-1-phenethyl-2,5-dihydro-1*H*-pyrrole-2-carboxamide (7g):** Yellow solid (51% yield). ^1H NMR (400 MHz, DMSO-d_6) δ 11.23 (s, 1H, OH), 8.22 (d, J = 7.8 Hz, 1H, NH), 7.82 (d, J = 8.8 Hz, 2H, ArH), 7.35 – 7.27 (m, 2H, ArH), 7.22 (d, J = 7.4 Hz, 3H, ArH), 6.91 (d, J = 8.9 Hz, 2H, ArH), 4.54 (s, 1H, CH), 3.87 – 3.78 (m, 1H, CH), 3.75 (s, 3H, OCH_3), 3.62 (d, J = 4.2 Hz, 1H, CH_2), 3.09 – 2.98 (m, 1H, CH_2), 2.91 – 2.82 (m, 1H, CH_2), 2.78 – 2.64 (m, 1H, CH_2), 1.79 (d, J = 8.6 Hz, 2H, CH_2), 1.70 (d, J = 12.6 Hz, 2H, CH_2), 1.57 (d, J = 12.1 Hz, 1H, CH_2), 1.32 – 1.22 (m, 5H, CH_2). ^{13}C NMR (100 MHz, DMSO-d_6) δ 171.80, 165.11, 164.05, 157.88, 139.66, 128.94, 128.91, 126.67, 124.71, 113.69, 104.84, 63.65, 55.45, 48.71, 41.93, 34.41, 32.82, 32.43, 25.65, 24.86. HRMS: m/z calculated for $C_{26}H_{31}N_2O_4$ $[M+H]^+$: 435.22783, found 435.22805. The analytical data matched those reported in the literature[2].

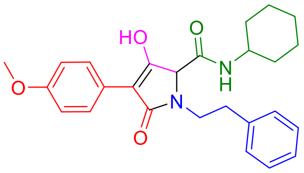

***N*-Cyclohexyl-1-(2,6-dimethylphenyl)-3-hydroxy-4-(4-nitrophenyl)-5-oxo-2,5-dihydro-1*H*-pyrrole-2-carboxamide (7h):** Yellow solid (75% yield). ^1H NMR (400 MHz, DMSO-d_6) δ 8.37 (d, J = 9.1 Hz, 2H, ArH), 8.27 – 8.17 (m, 2H, ArH), 7.97 (d, J = 7.7 Hz, 1H, NH), 7.25 – 7.03 (m, 3H, ArH), 4.80 (s, 1H,

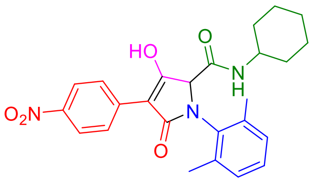

CH), 3.48-3.46 (m, 1H, CH), 2.14 (s, 3H, CH_3), 2.12 (s, 3H, CH_3), 1.80 – 1.64 (m, 2H, CH_2), 1.54 – 1.38 (m, 2H, CH_2), 1.26 – 1.11 (m, 4H, CH_2), 1.09 – 0.98 (m, 1H, CH_2)), 0.85 – 0.71 (m, 1H, CH_2). ^{13}C NMR (100 MHz, DMSO-d_6) δ 170.46, 164.54, 144.76, 140.23, 139.53, 137.45, 135.28, 128.59, 128.22, 127.29, 123.69, 102.27, 64.82, 48.57, 32.33, 25.55, 24.78, 18.48, 18.25. HRMS: m/z calculated for $C_{25}H_{28}N_3O_5$ $[M+H]^+$: 450.20235, found 450.20279. The analytical data matched those reported in the literature [2].

4-(2-Bromophenyl)-3-hydroxy-5-oxo-*N*,1-diphenyl-2,5-dihydro-1*H*-pyrrole-2-carboxamide (7i): Yellow solid (67% yield). ^{1}H NMR (400 MHz, DMSO-d_6) δ 11.93 (s, 1H, OH), 10.64 (s, 1H, NH), 7.72 – 7.56 (m, 5H, ArH), 7.44 (t, J = 7.3 Hz, 1H, ArH), 7.43 – 7.28 (m, 6H, ArH), 7.17 – 6.98 (m, 2H, ArH), 5.54 (s, 1H, CH). ^{13}C NMR (100 MHz, DMSO-d_6) δ 170.21, 165.66, 139.07, 138.81, 133.58, 132.75, 132.14, 130.12, 129.38, 129.36, 127.72, 125.44, 124.54, 123.56, 119.79, 119.24, 107.77, 64.53. HRMS: m/z calculated for $C_{23}H_{18}BrN_2O_3$ $[M+H]^+$: 449.04953, found 449.04910. The analytical data matched those reported in the literature[2].

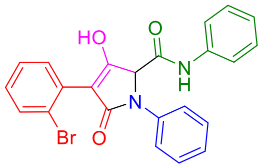

4-(4-Chlorophenyl)-3-hydroxy-1-isobutyl-5-oxo-*N*-phenyl-2,5-dihydro-1*H*-pyrrole-2-carboxamide (7j): Yellow solid (53% yield). ^{1}H NMR (400 MHz, DMSO-d_6) δ 11.99 (s, 1H, OH), 10.40 (s, 1H, NH), 8.00 (d, J = 8.6 Hz, 2H, ArH), 7.65 (d, J = 7.9 Hz, 2H, ArH), 7.45 – 7.33 (m, 4H, ArH), 7.13 (d, J = 7.4 Hz, 1H, ArH), 4.79 (s, 1H, CH), 3.39 – 3.33 (m, 1H, CH_2), 2.81 (dd, J = 13.8, 6.4 Hz, 1H, CH_2), 2.03 – 1.86 (m, 1H, CH), 0.86 (d, J = 6.6 Hz, 3H, CH_3), 0.80 (d, J = 6.6 Hz, 3H, CH_3). ^{13}C NMR (100 MHz, DMSO-d_6) δ 171.86, 165.95, 164.98, 138.85, 131.23, 130.76, 129.34, 129.22, 128.27, 124.60, 120.10, 103.87, 64.97, 48.23, 27.48, 20.63, 20.39. HRMS: m/z calculated for $C_{21}H_{22}ClN_2O_3$ $[M+H]^+$: 385.13135, found 385.13074. The analytical data matched those reported in the literature[2].

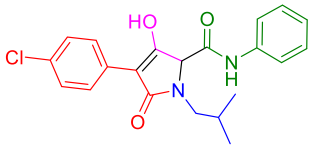

1-Benzyl-4-(4-bromophenyl)-*N*-(2,6-dimethylphenyl)-3-hydroxy-5-oxo-2,5-di

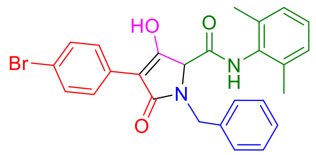

-hydro-1*H*-pyrrole-2-carboxamide (7k): Yellow solid (63% yield).^{1}H NMR (400 MHz, DMSO-d_6) δ 10.27 (s, 1H, OH), 9.50 (s, 1H, NH), 8.44 (d, J = 8.6 Hz, 2H, ArH), 7.32 (t, J = 7.2 Hz, 2H, ArH), 7.21 (dd, J = 18.8, 8.0 Hz, 5H, ArH), 7.03 (s, 3H, ArH), 5.23 (d, J = 15.2 Hz, 1H, CH_2), 3.94 (s, 1H, CH), 3.89 (d, J = 15.2 Hz, 1H CH_2), 2.13 (s, 6H, $ArCH_3$). ^{13}C NMR (100 MHz, DMSO-d_6) δ 182.44, 176.42, 167.45, 139.76, 138.30, 135.75, 135.55, 130.00, 128.83, 128.18, 127.92, 127.13, 126.51, 125.07, 112.18, 90.05, 65.26, 44.23, 18.66. HRMS: m/z calculated for $C_{26}H_{24}BrN_2O_3$ $[M+H]^+$: 491.09648, found 491.09662.

4-(Benzo[d][1,3]dioxol-5-yl)-*N*-(2,6-dimethylphenyl)-3-hydroxy-5-oxo-1-phenyl-2,5-dihydro-1*H*-pyrrole-2-carboxamide(7l): Yellow solid (71% yield).^{1}H

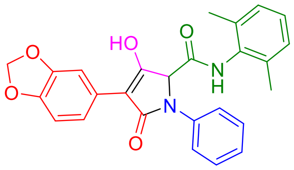

NMR (400 MHz, DMSO-d_6) δ 10.32 (s, 1H, OH), 9.67 (s, 1H, NH), 8.15 (s, 1H, ArH), 8.01 (d, J = 8.9 Hz, 1H, ArH), 7.73 (d, J = 8.1 Hz, 2H, ArH), 7.19 (t, J = 7.8 Hz, 2H, ArH), 7.00 (s, 3H, ArH), 6.82 (t, J = 7.2 Hz, 1H, ArH), 6.71 (d, J = 8.3 Hz, 1H, ArH), 5.86 (s, 2H, OCH_2O), 4.81 (s, 1H, CH), 2.13 (s, 6H, $ArCH_3$). ^{13}C NMR (100 MHz, DMSO-d_6) δ 181.13, 175.23, 167.35, 146.41, 141.84, 141.40, 135.90, 135.68, 133.08, 128.51, 127.89, 126.52, 120.13, 117.96, 116.52, 107.62, 104.67, 66.66, 18.71. HRMS: m/z calculated for $C_{26}H_{23}N_2O_5$ $[M+H]^+$: 443.16015, found 443.16035.

References

[1] Li, Y.; Lei, J.; Chen, Z.; Tang, D.; Yuan, H.; Wang, M.; Zhu, J.; Xu, Z. *Eur. J. Org. Chem.* **2016**, 2016, 5770-5776.

[2] Chen, Z.; Xu, Z. CN 000107434778 A, 2017.12.05.

Copies of ^1H and ^{13}C NMR spectra

Compound **7a**

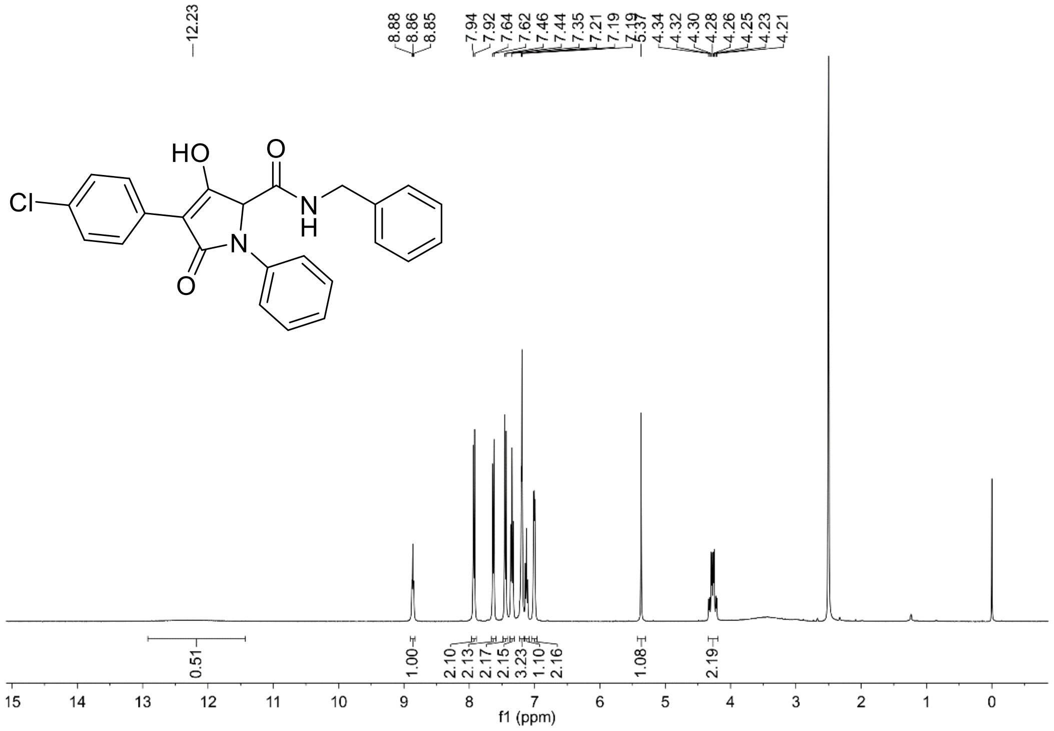

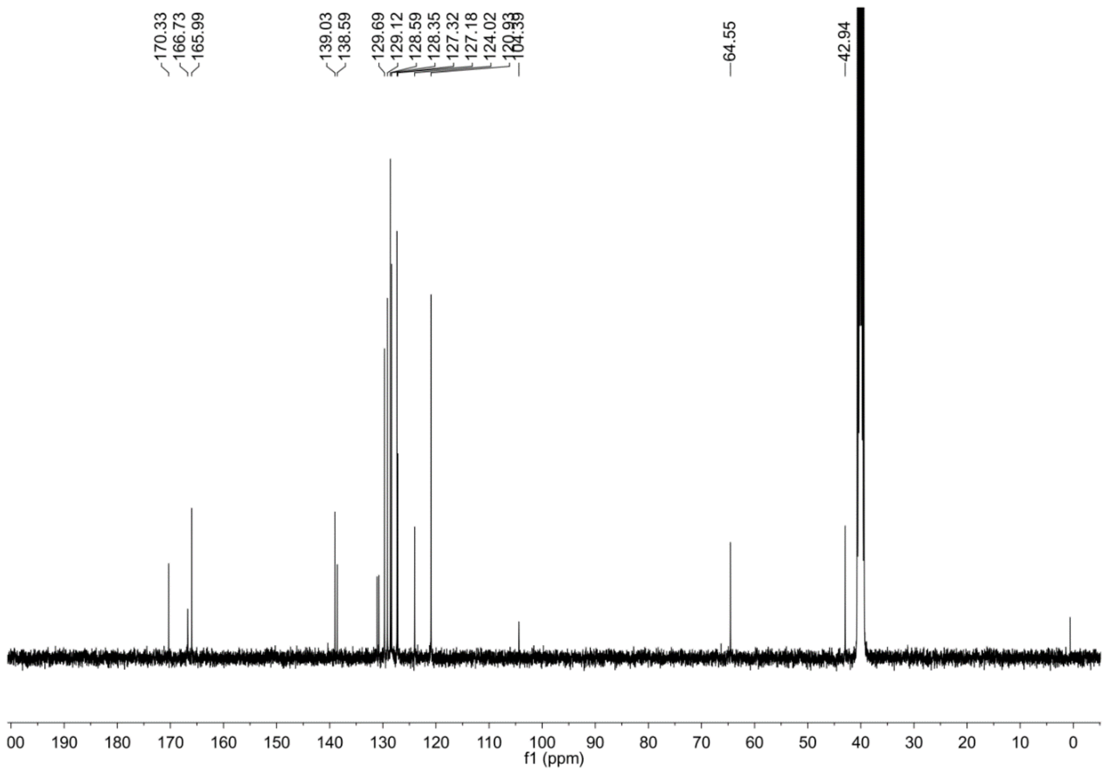

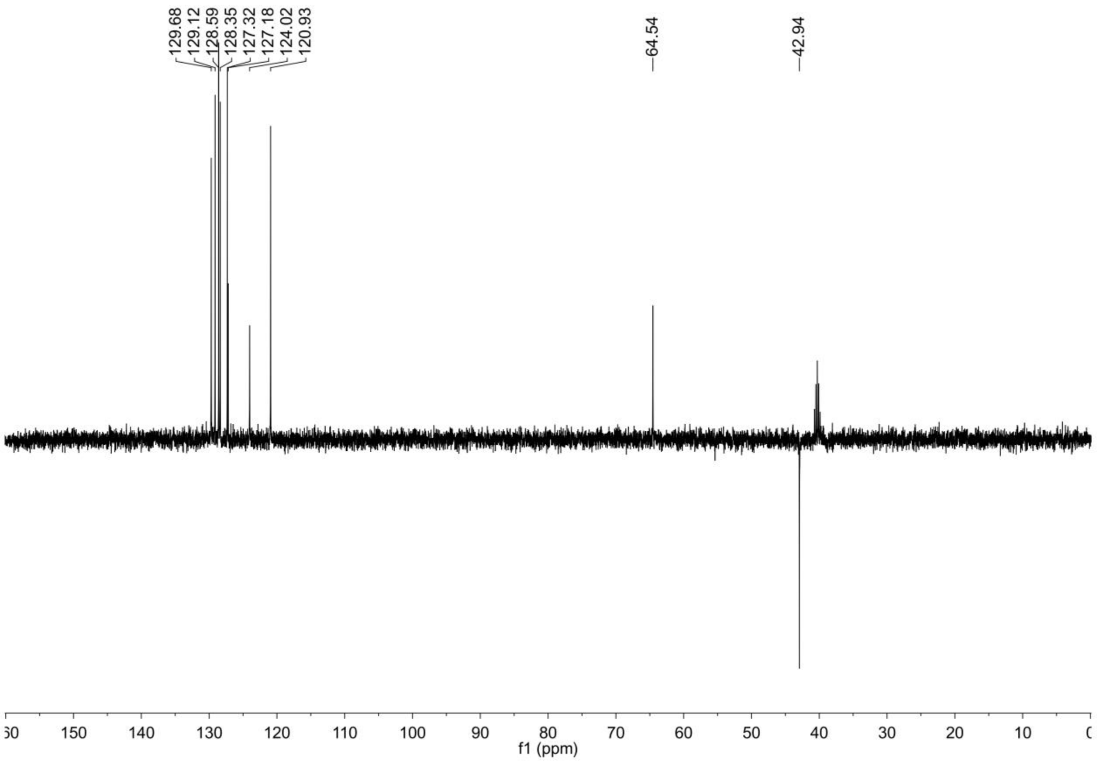

129.68
129.12
128.59
128.35
127.32
127.18
124.02
120.93
64.54
42.94
150
140
130
120
110
100
90
80
70
60
50
40
30
20
10
f1 (ppm)

Compound **7b**

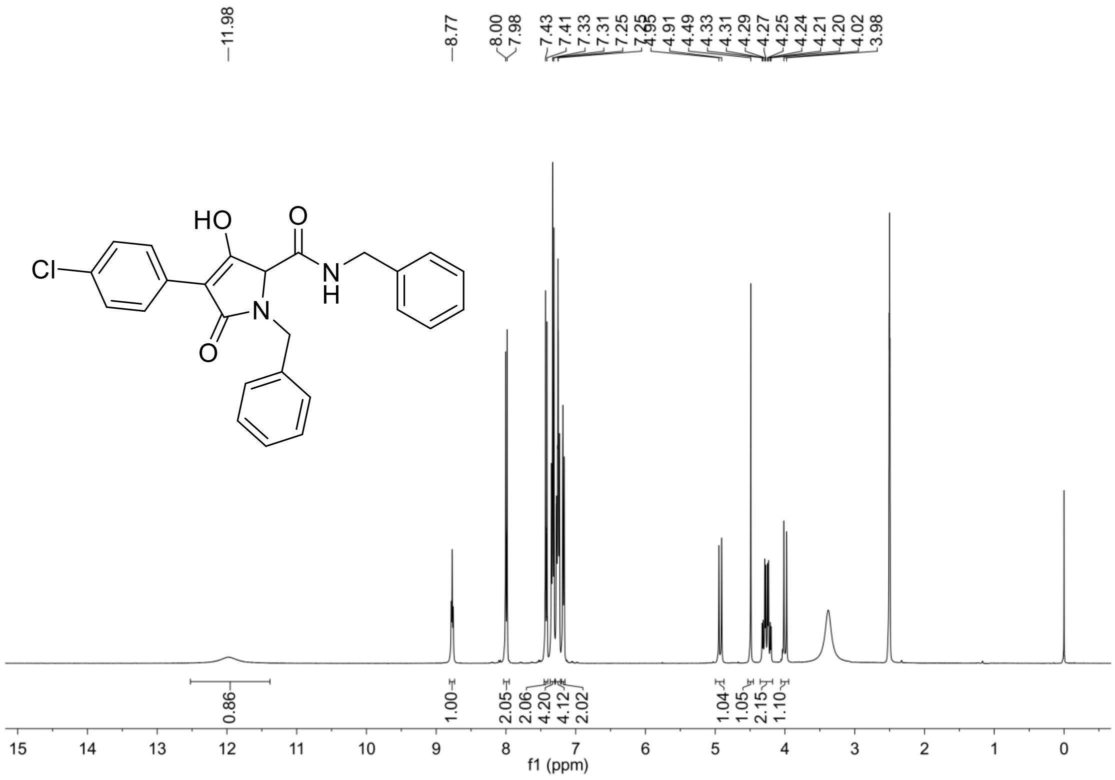
11.98
8.77
8.00
7.98
7.43
7.41
7.33
7.31
7.25
4.95
4.91
4.49
4.33
4.31
4.29
4.27
4.25
4.24
4.21
4.20
4.02
3.98
HO
O
Cl
N
H
O
0.86
1.00
2.05
2.06
4.20
4.12
2.02
1.04
1.05
2.15
1.10
f1 (ppm)

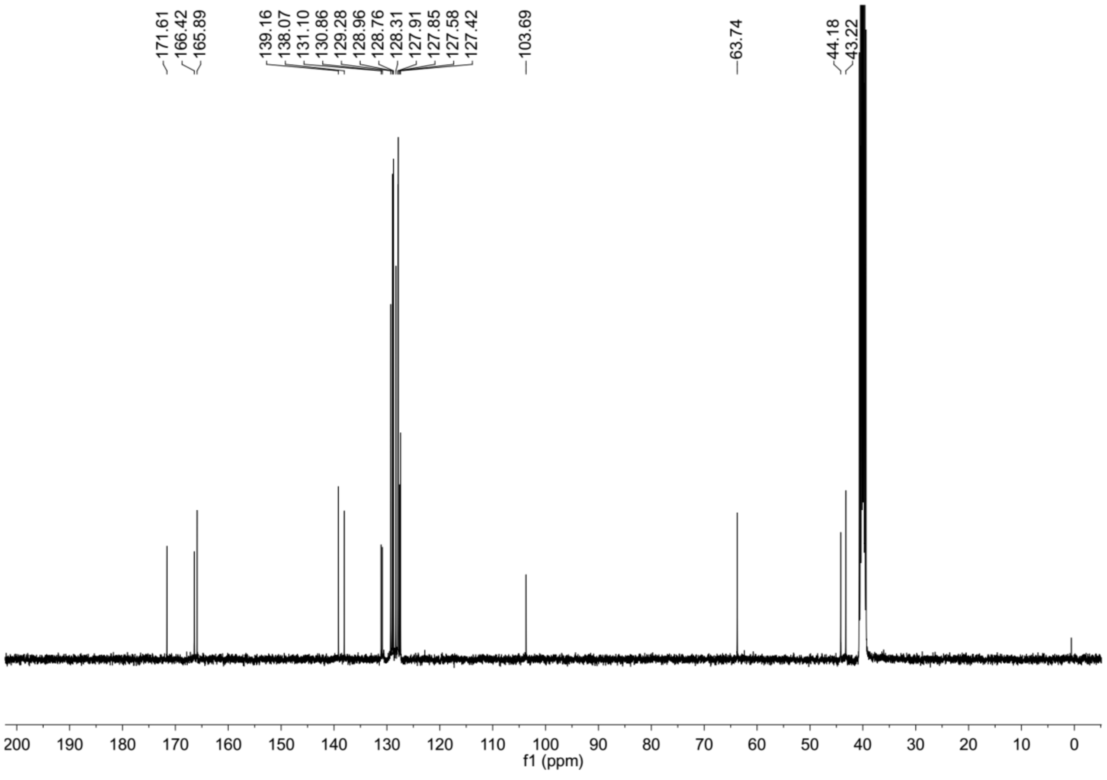
171.61
166.42
165.89
139.16
138.07
131.10
130.86
129.28
128.96
128.76
128.31
127.91
127.85
127.58
127.42
103.69
63.74
44.18
43.22
f1 (ppm)

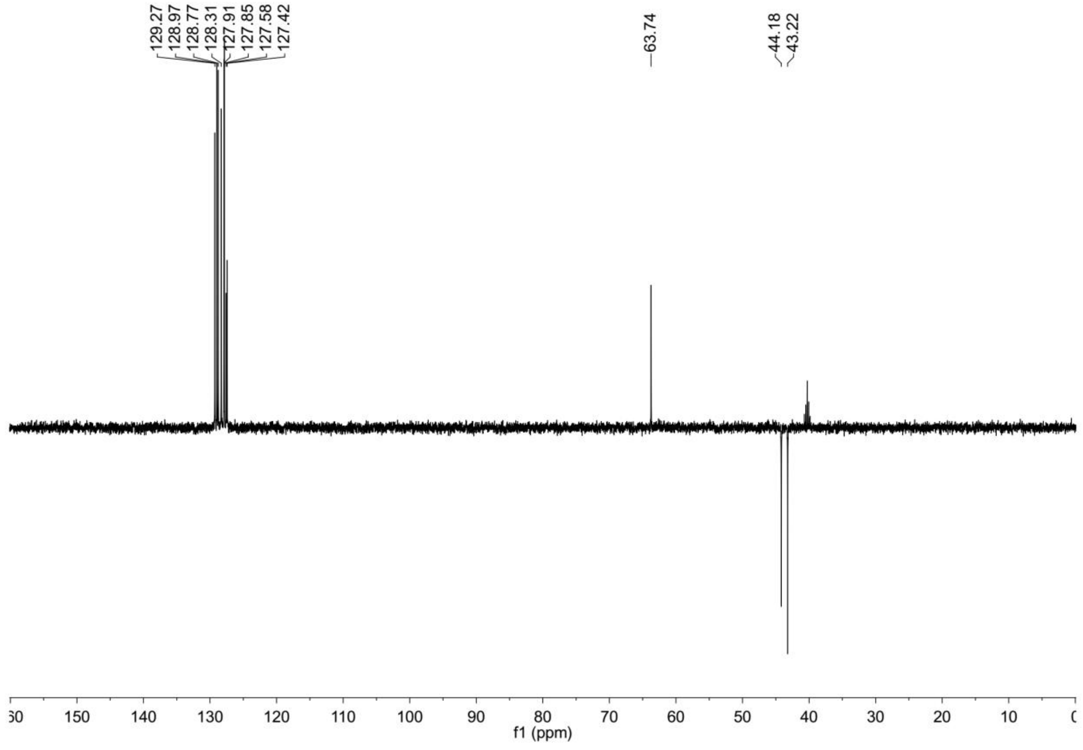
129.27
128.97
128.77
128.31
127.91
127.85
127.58
127.42
63.74
44.18
43.22
150
140
130
120
110
100
90
80
70
60
50
40
30
20
10
f1 (ppm)

Compound **7c**

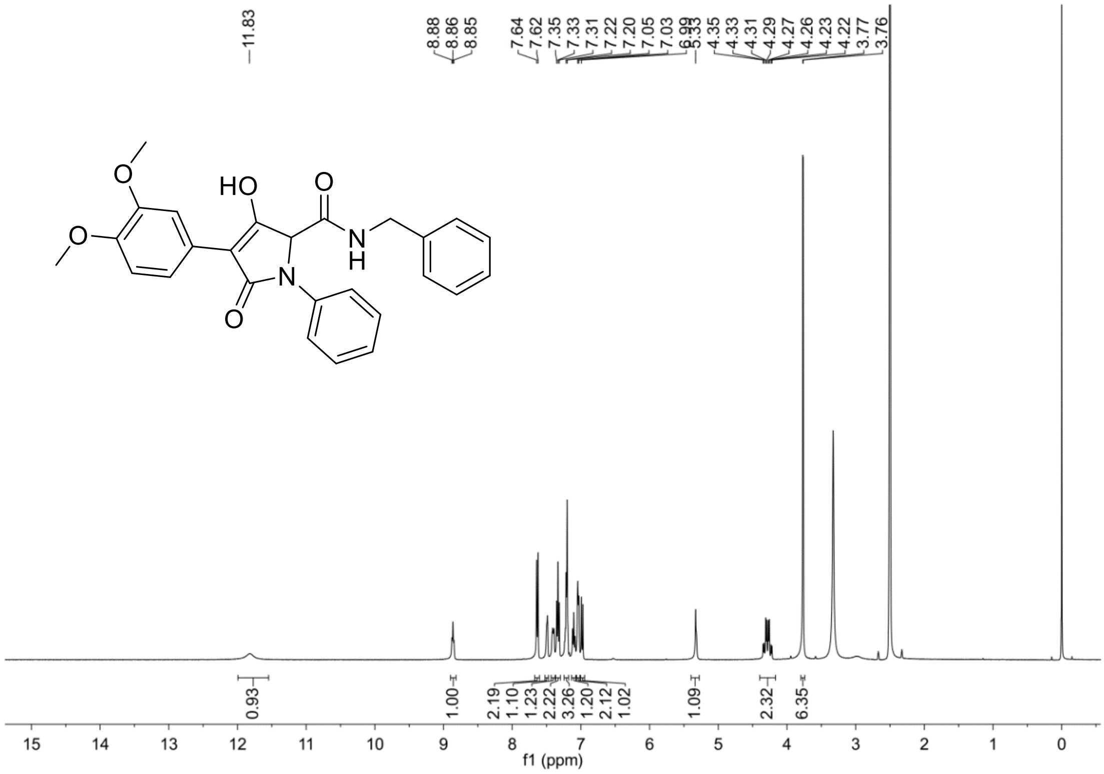
O
HO
O
N
H
N
O
O
11.83
8.88
8.86
8.85
7.64
7.62
7.35
7.33
7.31
7.22
7.20
7.05
7.03
6.99
5.33
4.35
4.33
4.31
4.29
4.27
4.26
4.23
4.22
3.77
3.76
0.93
1.00
2.19
1.10
1.23
2.22
3.26
1.20
2.12
1.02
1.09
2.32
6.35
15 14 13 12 11 10 9 8 7 6 5 4 3 2 1 0
f1 (ppm)

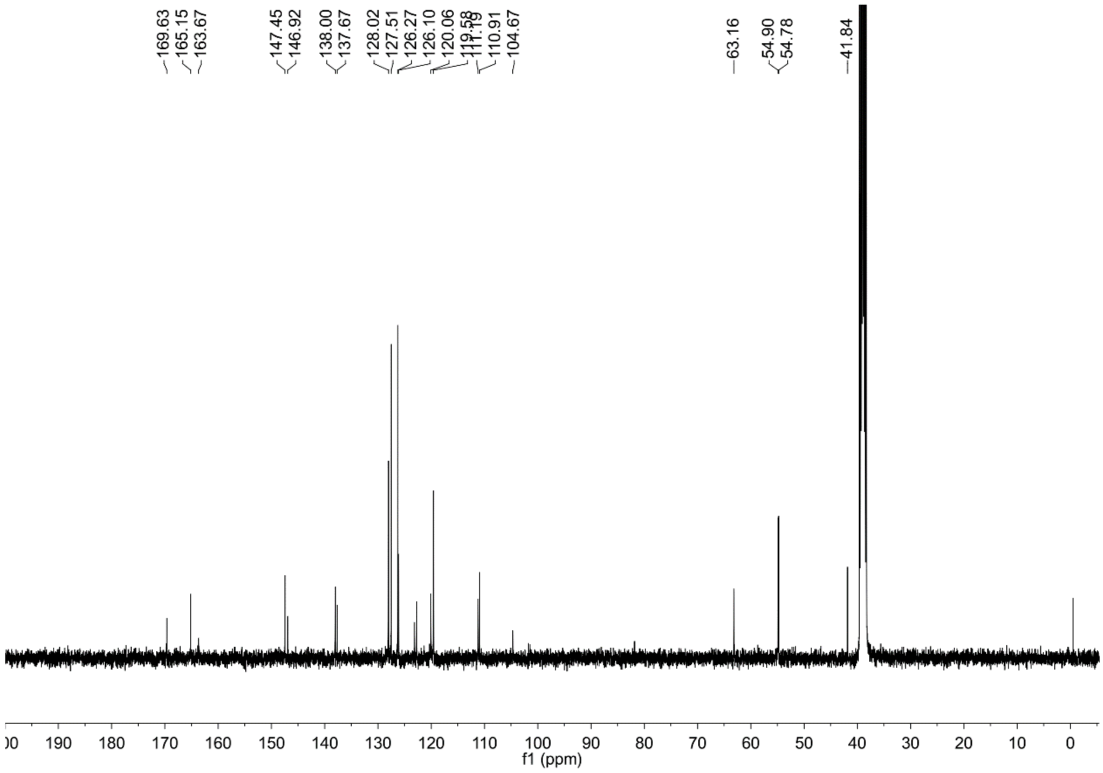
169.63
165.15
163.67
147.45
146.92
138.00
137.67
128.02
127.51
126.27
126.10
120.06
119.58
111.19
110.91
104.67
63.16
54.90
54.78
41.84
190 180 170 160 150 140 130 120 110 100 90 80 70 60 50 40 30 20 10 0
f1 (ppm)

Compound **7d**

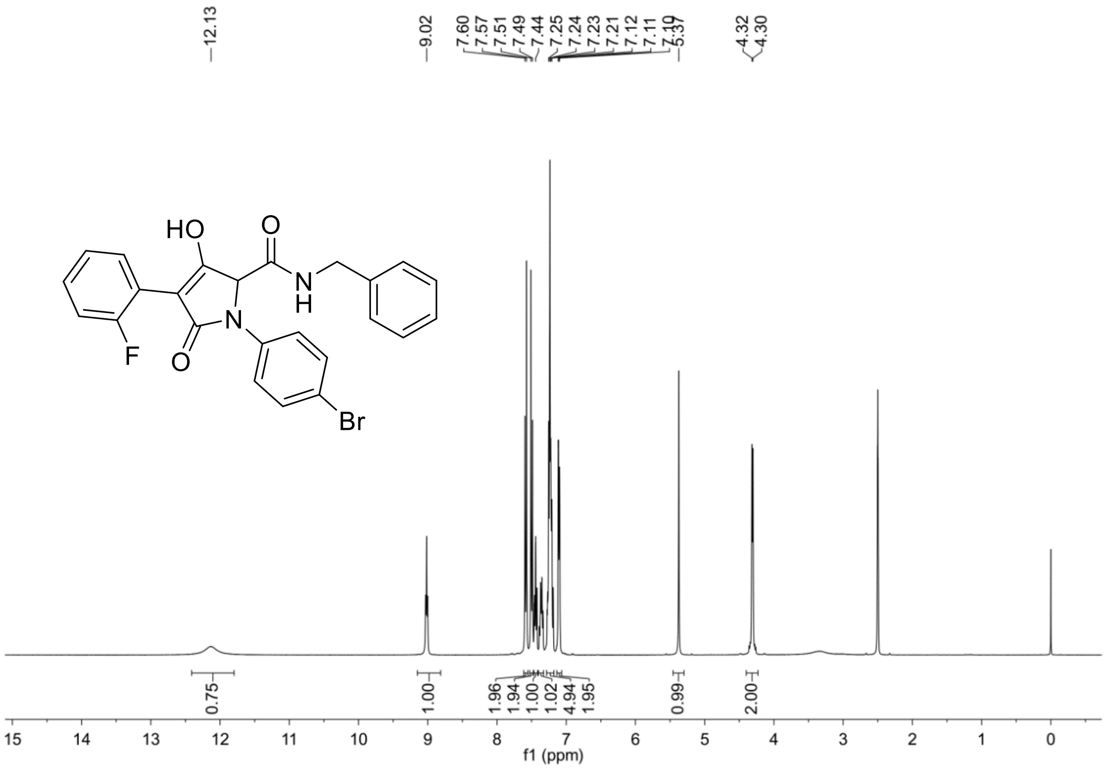

HO
O
N
H
F
O
N
Br
12.13
9.02
7.60
7.57
7.51
7.49
7.44
7.25
7.24
7.23
7.21
7.12
7.11
7.10
5.37
4.32
4.30
0.75
1.00
1.96
1.94
1.00
1.02
4.94
1.95
0.99
2.00
f1 (ppm)

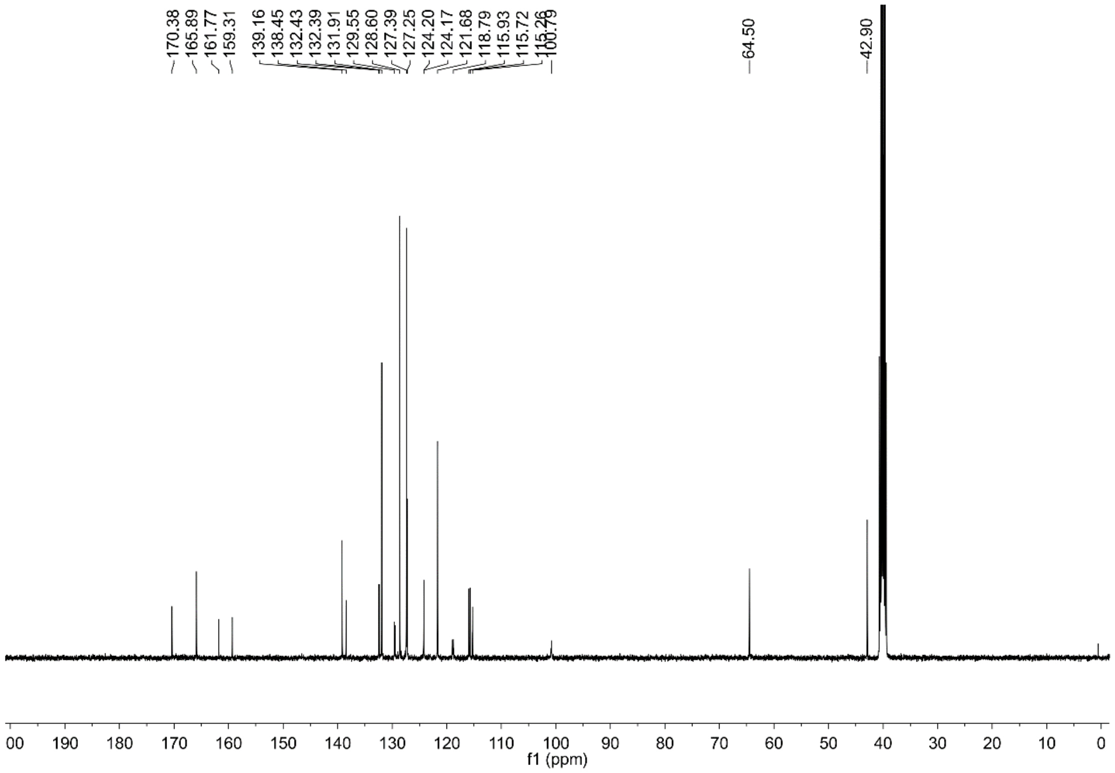

170.38
165.89
161.77
159.31
139.16
138.45
132.43
132.39
131.91
129.55
128.60
127.39
127.25
124.20
124.17
121.68
118.79
115.93
115.72
115.26
100.79
64.50
42.90
f1 (ppm)

Compound **7e**

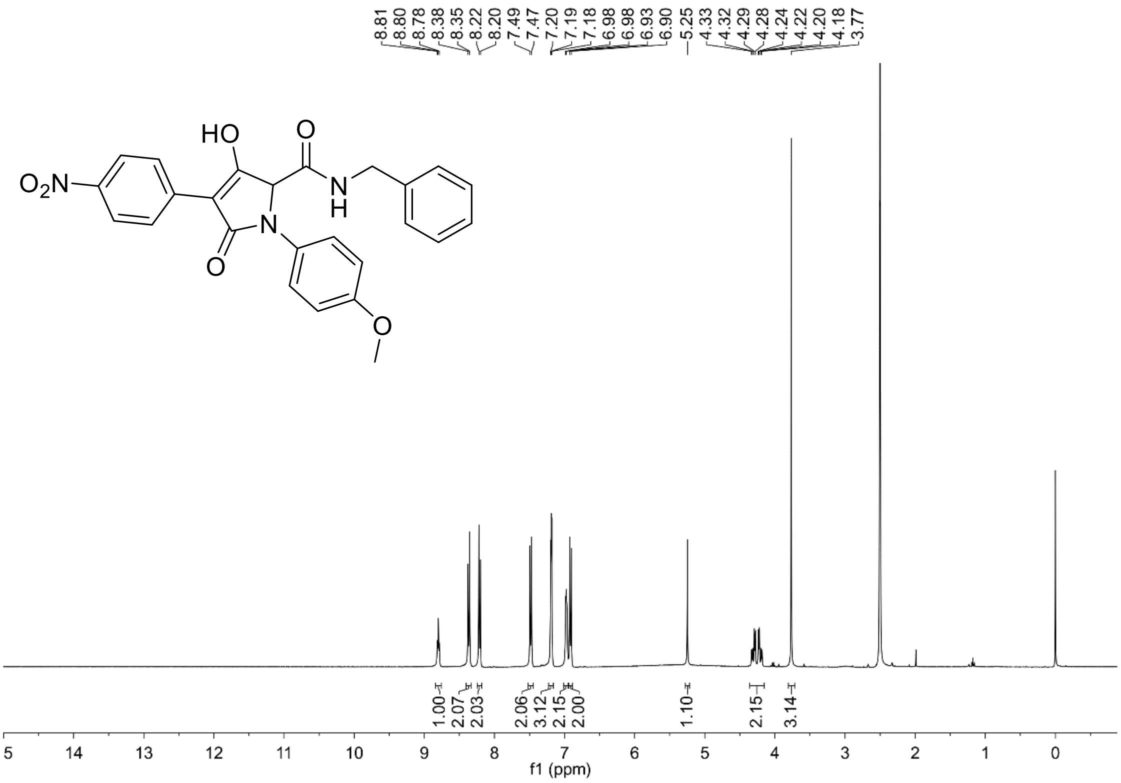

8.81
8.80
8.78
8.38
8.35
8.22
8.20
7.49
7.47
7.20
7.19
7.18
6.98
6.98
6.93
6.90
5.25
4.33
4.32
4.29
4.28
4.24
4.22
4.20
4.18
3.77
HO
O
O_2N
N
H
N
O
O
1.00
2.07
2.03
2.06
3.12
2.15
2.00
1.10
2.15
3.14
f1 (ppm)

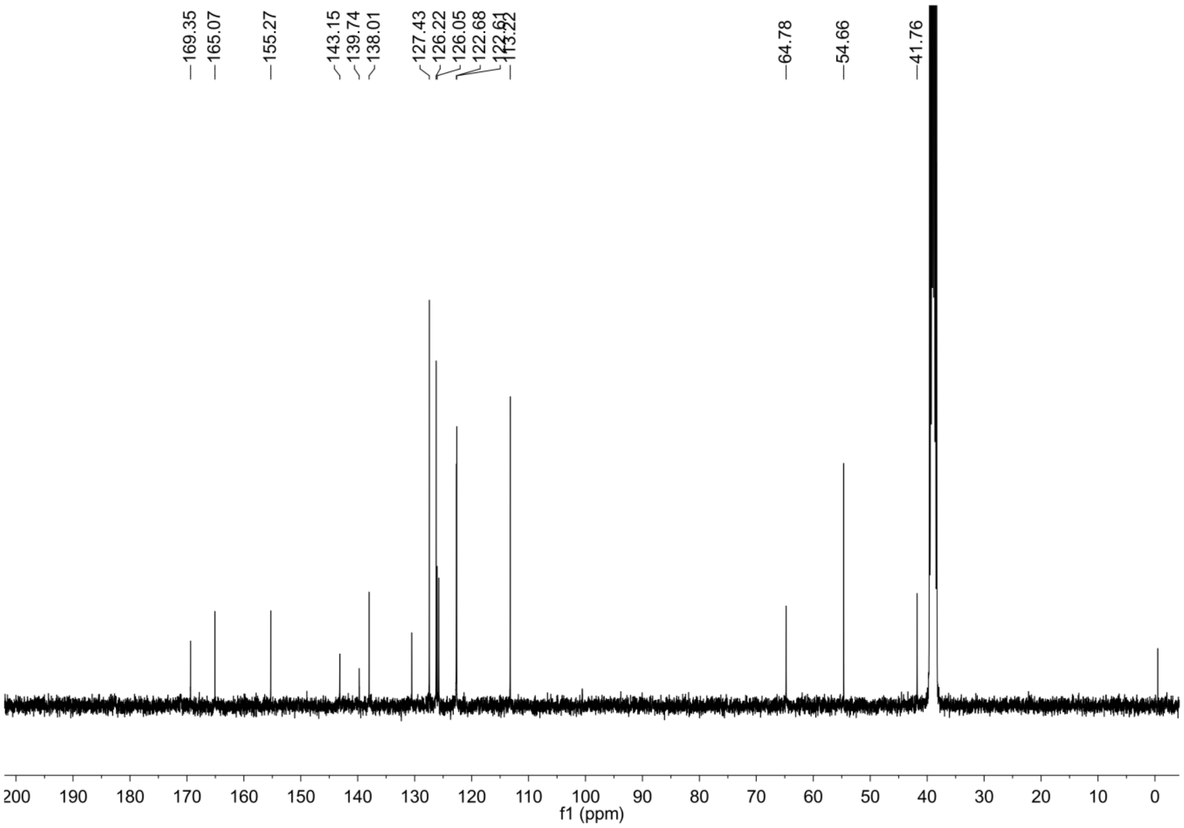

169.35
165.07
155.27
143.15
139.74
138.01
127.43
126.22
126.05
122.68
113.22
64.78
54.66
41.76
f1 (ppm)

Compound **7f**

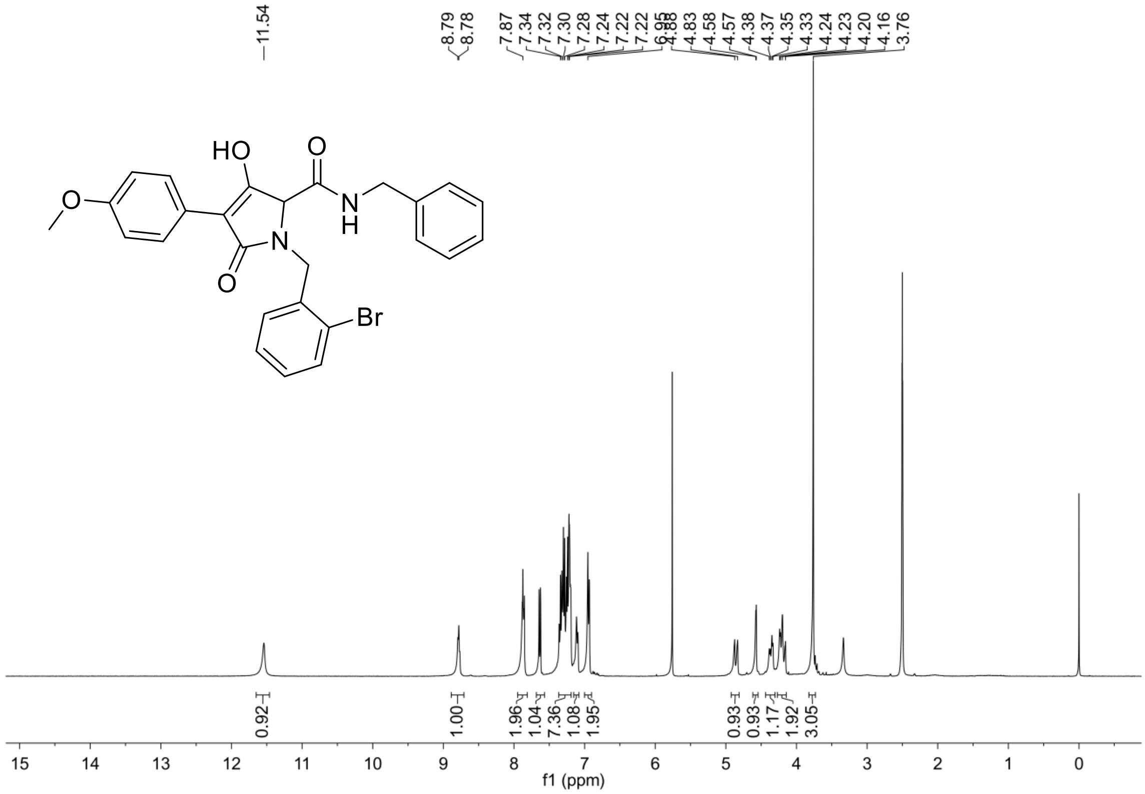

11.54
8.79
8.78
7.87
7.34
7.32
7.30
7.28
7.24
7.22
7.22
6.95
4.88
4.83
4.58
4.57
4.38
4.37
4.35
4.33
4.24
4.23
4.20
4.16
3.76
HO
O
N
H
N
O
O
Br
0.92
1.00
1.96
1.04
7.36
1.08
1.95
0.93
0.93
1.17
1.92
3.05
f1 (ppm)

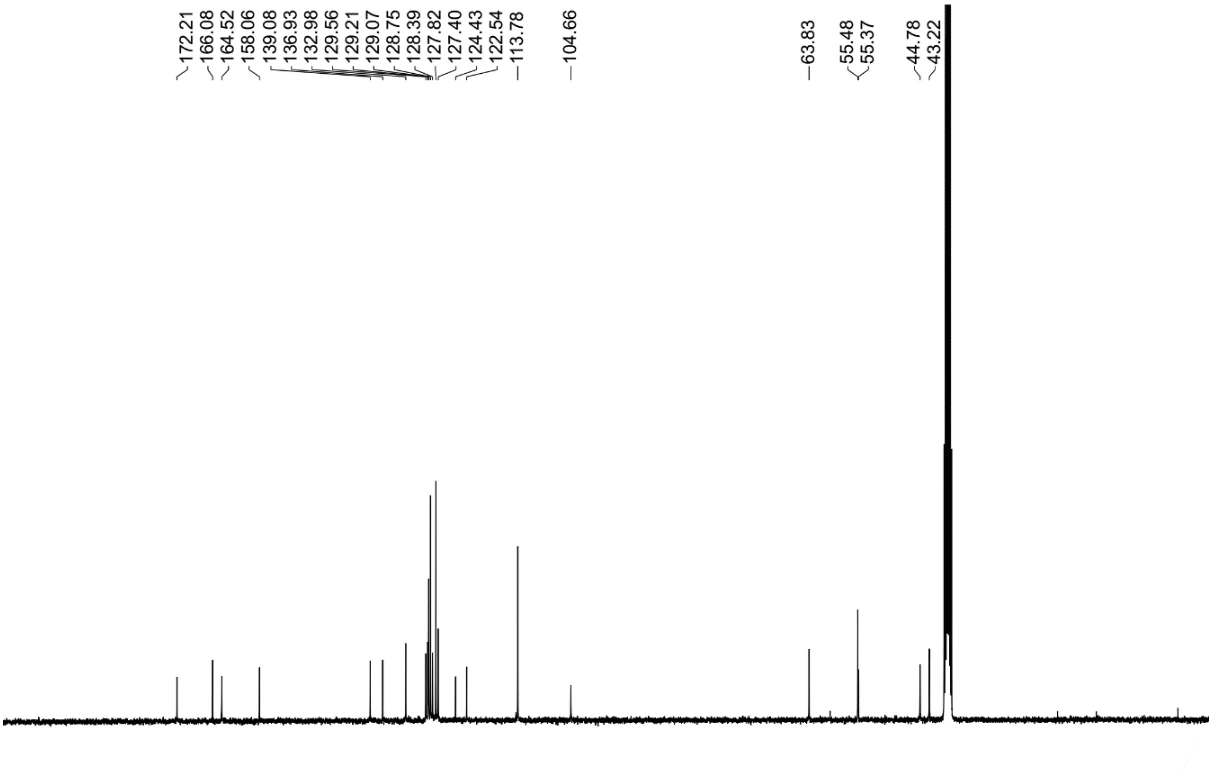

172.21
166.08
164.52
158.06
139.08
136.93
132.98
129.56
129.21
129.07
128.75
128.39
127.82
127.40
124.43
122.54
113.78
104.66
63.83
55.48
55.37
44.78
43.22
f1 (ppm)

Compound **7g**

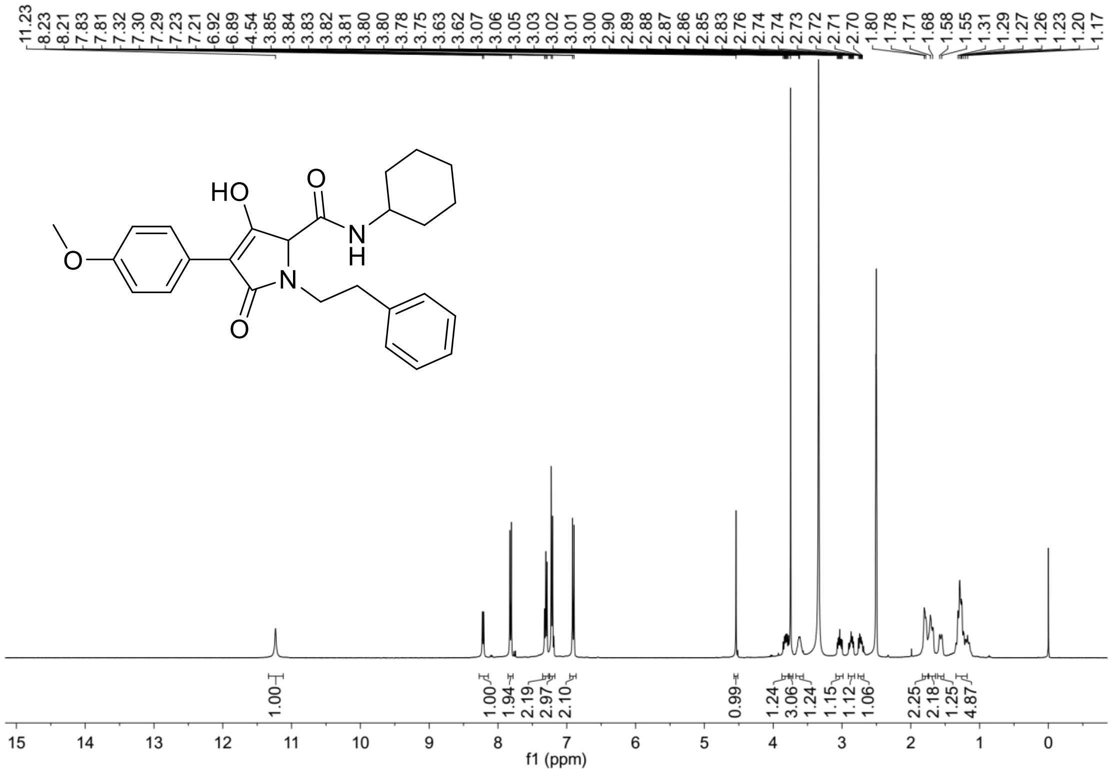
11.23 8.23 8.21 7.83 7.81 7.32 7.30 7.29 7.23 7.21 6.92 6.89 4.54 3.85 3.84 3.83 3.82 3.81 3.80 3.80 3.78 3.75 3.63 3.62 3.07 3.06 3.05 3.03 3.02 3.01 3.00 2.90 2.89 2.88 2.87 2.86 2.85 2.83 2.76 2.74 2.73 2.72 2.71 2.70 1.80 1.78 1.71 1.68 1.58 1.55 1.31 1.29 1.27 1.26 1.23 1.20 1.17
HO
O
N
H
O
N
O
1.00
1.00 1.94 2.19 2.97 2.10
0.99
1.24 3.06 1.24 1.15 1.12 1.06
2.25 2.18 1.25 4.87
15 14 13 12 11 10 9 8 7 6 5 4 3 2 1 0
f1 (ppm)

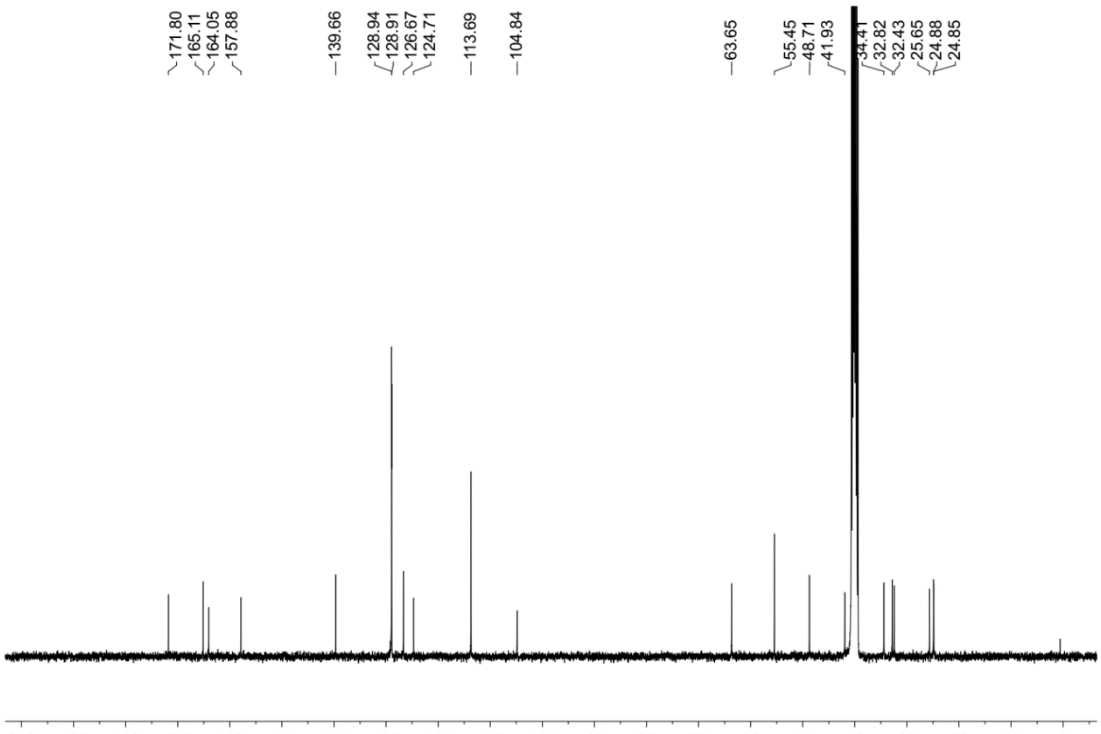
171.80 165.11 164.05 157.88
139.66
128.94 128.91 126.67 124.71
113.69
104.84
63.65
55.45 48.71 41.93
34.41 32.82 32.43 25.65 24.88 24.85
200 190 180 170 160 150 140 130 120 110 100 90 80 70 60 50 40 30 20 10 0
f1 (ppm)

Compound **7h**

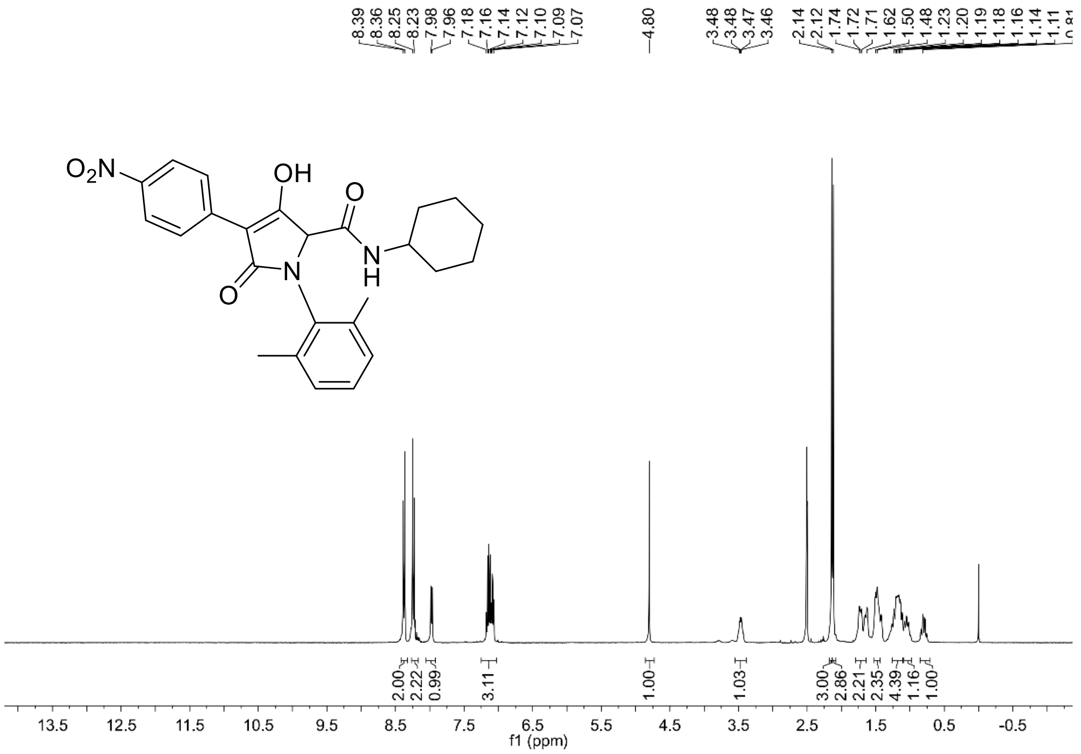

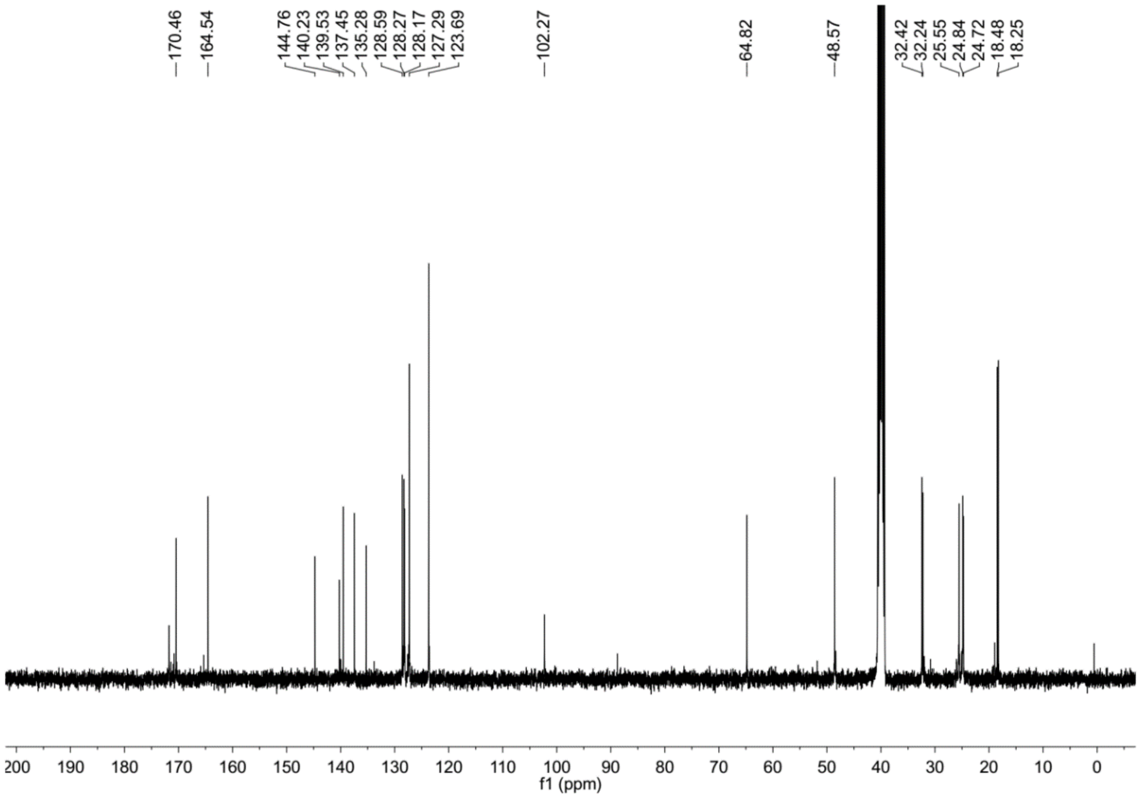

Compound **7i**

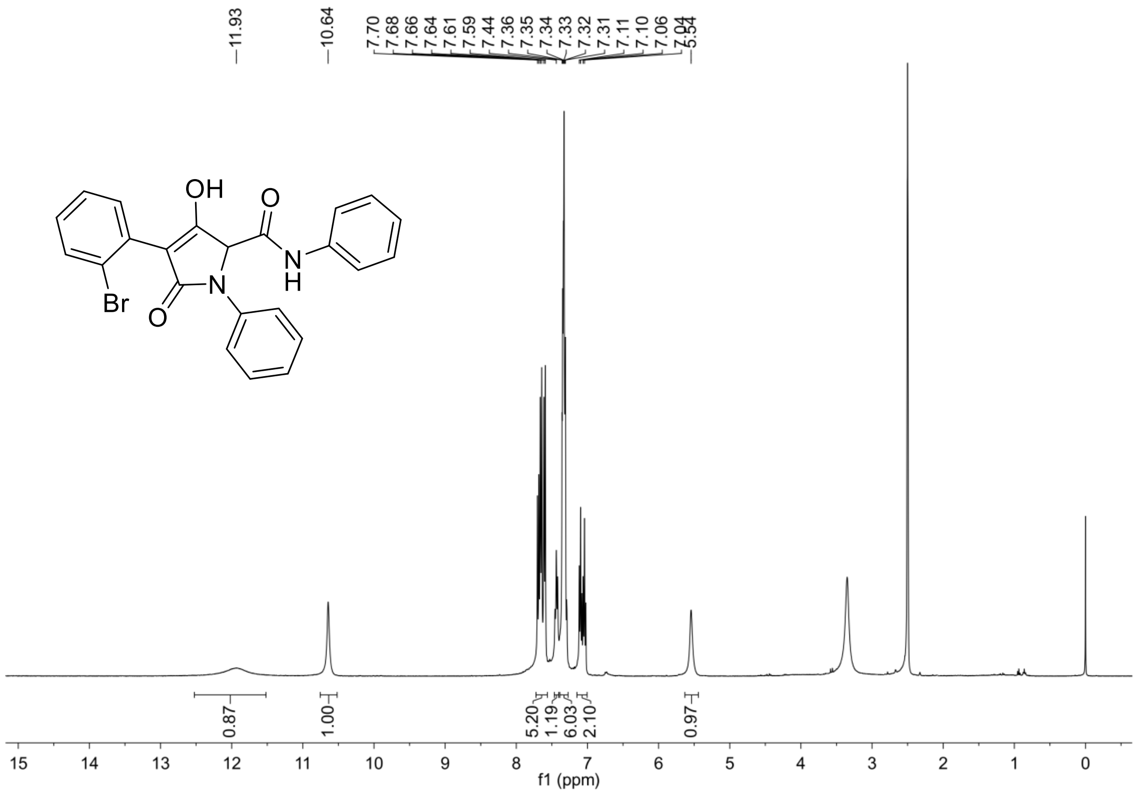

11.93
10.64
7.70
7.68
7.66
7.64
7.61
7.59
7.44
7.36
7.35
7.34
7.33
7.32
7.31
7.11
7.10
7.06
7.04
5.54
OH
O
N
H
Br
O
N
0.87
1.00
5.20
1.19
6.03
2.10
0.97
15 14 13 12 11 10 9 8 7 6 5 4 3 2 1 0
f1 (ppm)

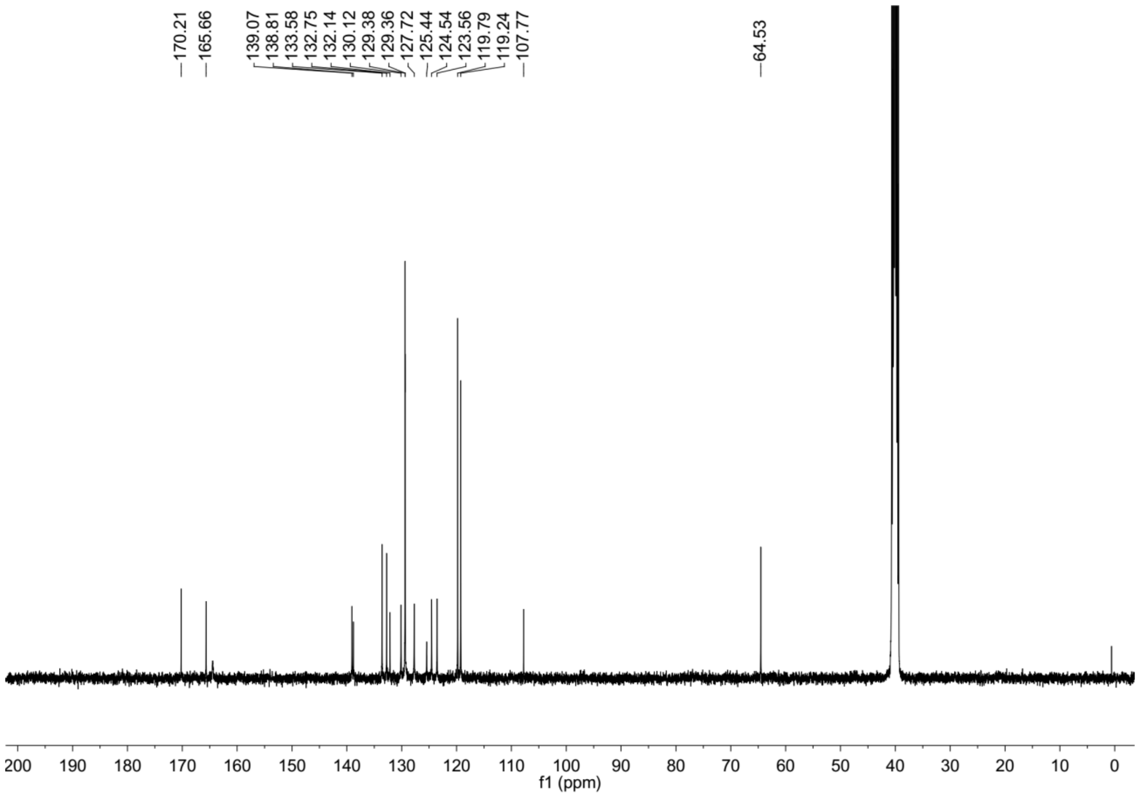

170.21
165.66
139.07
138.81
133.58
132.75
132.14
130.12
129.38
129.36
127.72
125.44
124.54
123.56
119.79
119.24
107.77
64.53
200 190 180 170 160 150 140 130 120 110 100 90 80 70 60 50 40 30 20 10 0
f1 (ppm)

Compound **7j**

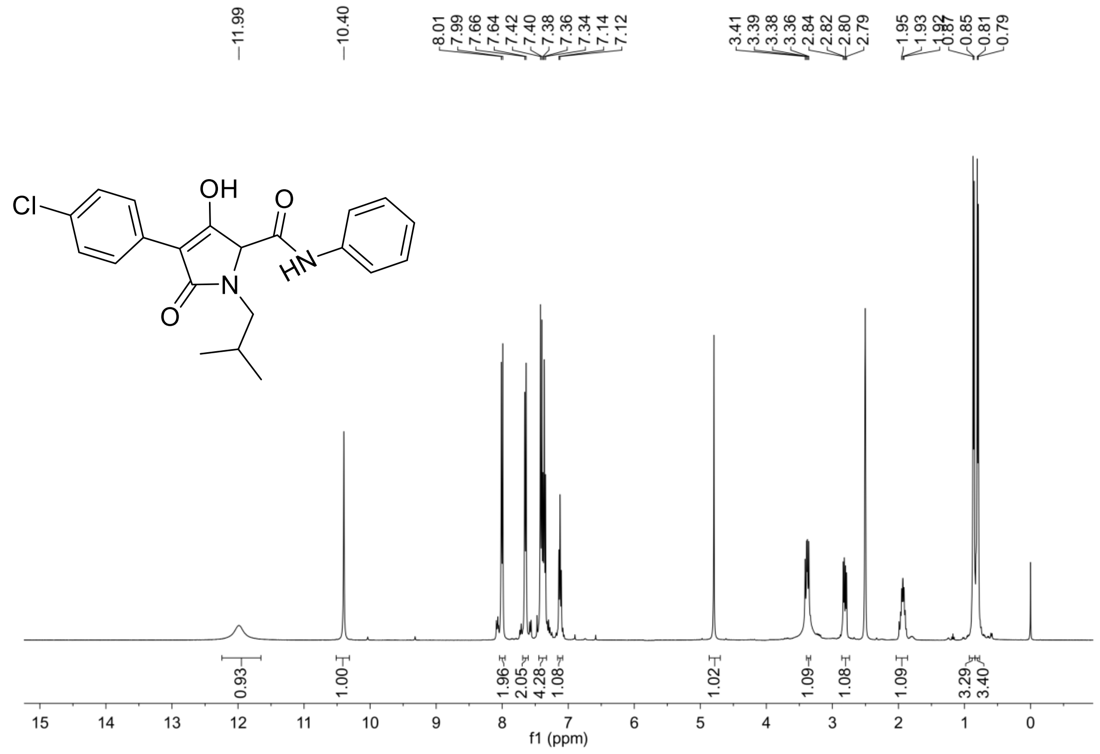
11.99
10.40
8.01
7.99
7.66
7.64
7.42
7.40
7.38
7.36
7.34
7.14
7.12
3.41
3.39
3.38
3.36
2.84
2.82
2.80
2.79
1.95
1.93
0.85
0.81
0.79
Cl
OH
O
N
HN
O
0.93
1.00
1.96
2.05
4.28
1.08
1.02
1.09
1.08
1.09
3.29
3.40
15
14
13
12
11
10
9
8
7
6
5
4
3
2
1
0
f1 (ppm)

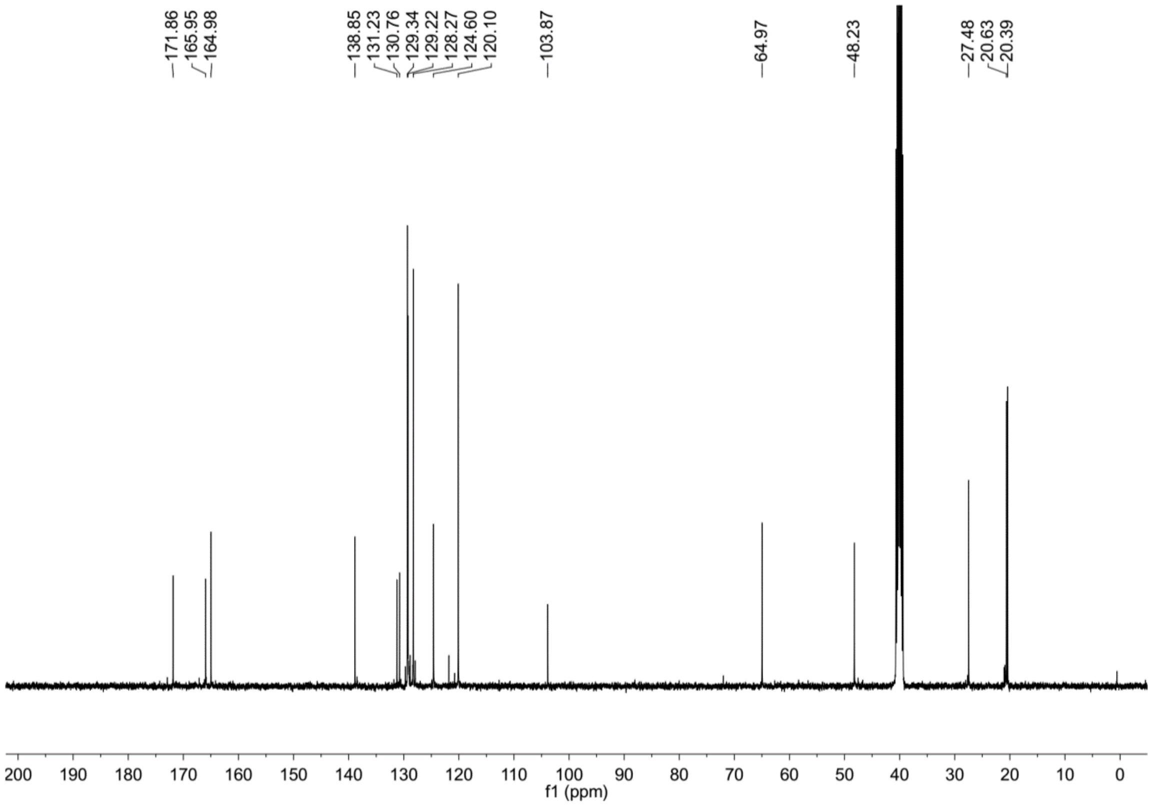
171.86
165.95
164.98
138.85
131.23
130.76
129.34
129.22
128.27
124.60
120.10
103.87
64.97
48.23
27.48
20.63
20.39
200
190
180
170
160
150
140
130
120
110
100
90
80
70
60
50
40
30
20
10
0
f1 (ppm)

Compound **7k**

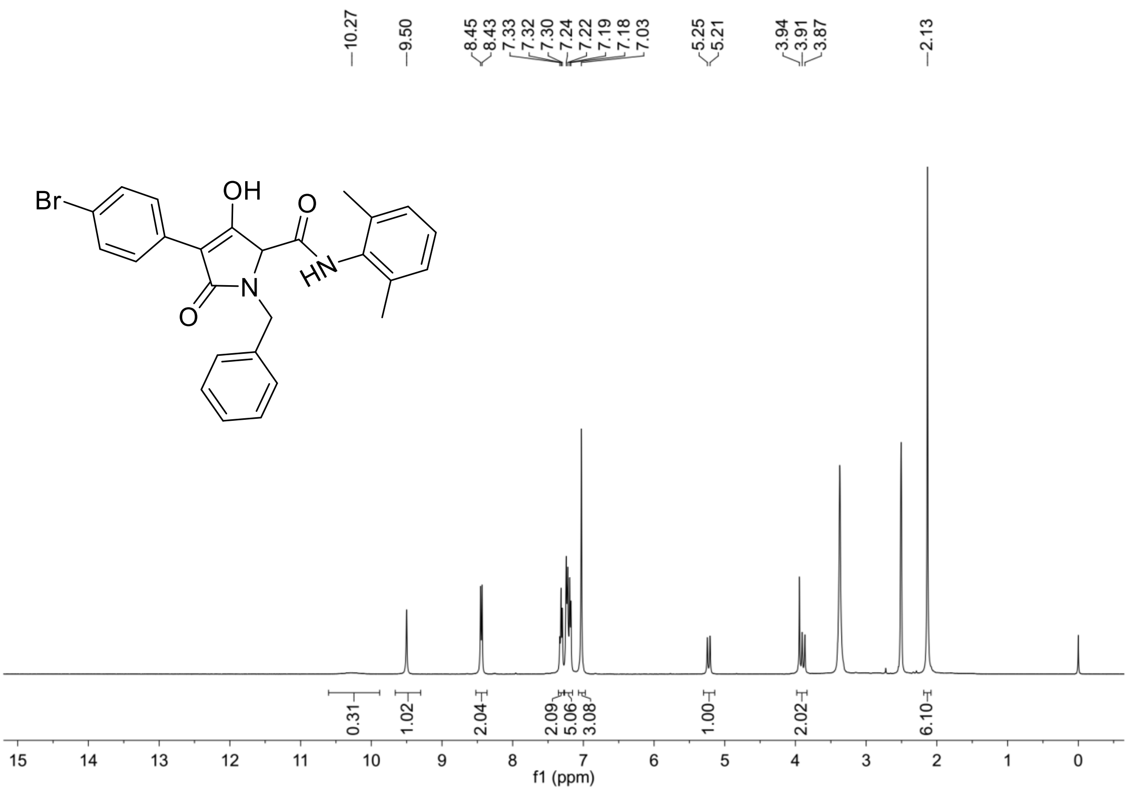
10.27
9.50
8.45
8.43
7.33
7.32
7.30
7.24
7.22
7.19
7.18
7.03
5.25
5.21
3.94
3.91
3.87
2.13
Br
OH
O
HN
O
N
0.31
1.02
2.04
2.09
5.06
3.08
1.00
2.02
6.10
15
14
13
12
11
10
9
8
7
6
5
4
3
2
1
0
f1 (ppm)

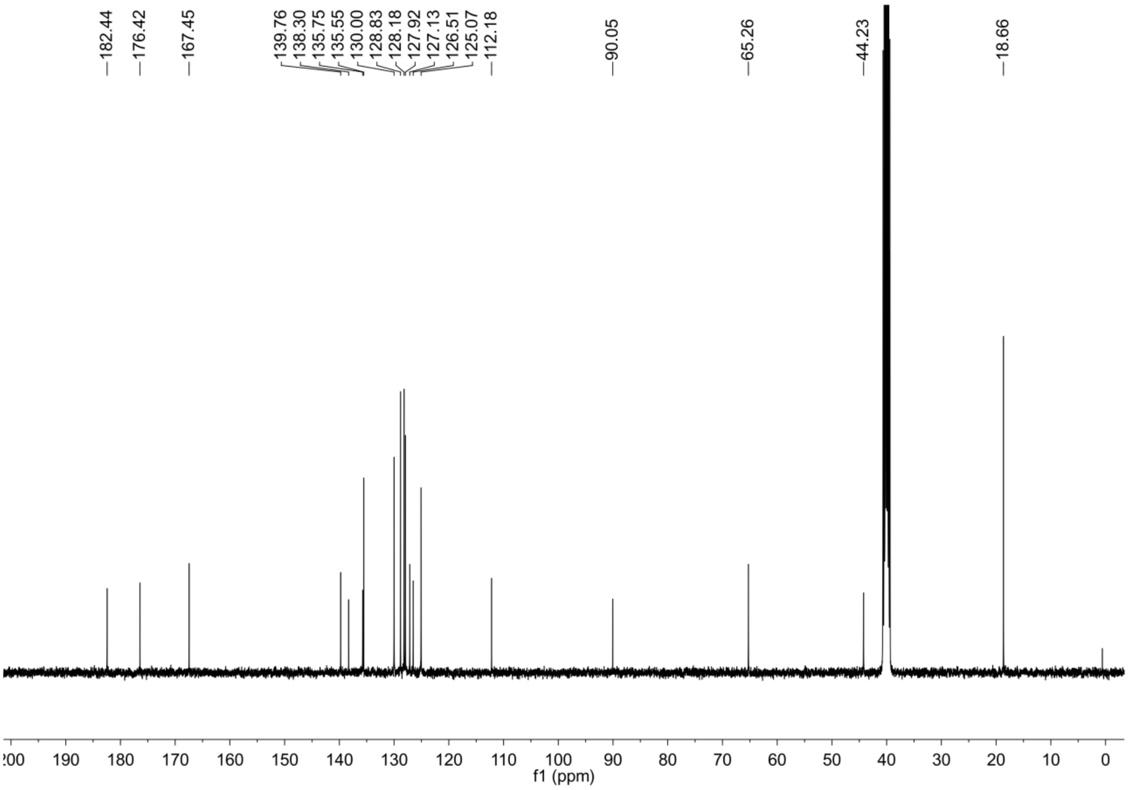
182.44
176.42
167.45
139.76
138.30
135.75
135.55
130.00
128.83
128.18
127.92
127.13
126.51
125.07
112.18
90.05
65.26
44.23
18.66
190
180
170
160
150
140
130
120
110
100
90
80
70
60
50
40
30
20
10
0
f1 (ppm)

Compound **7l**

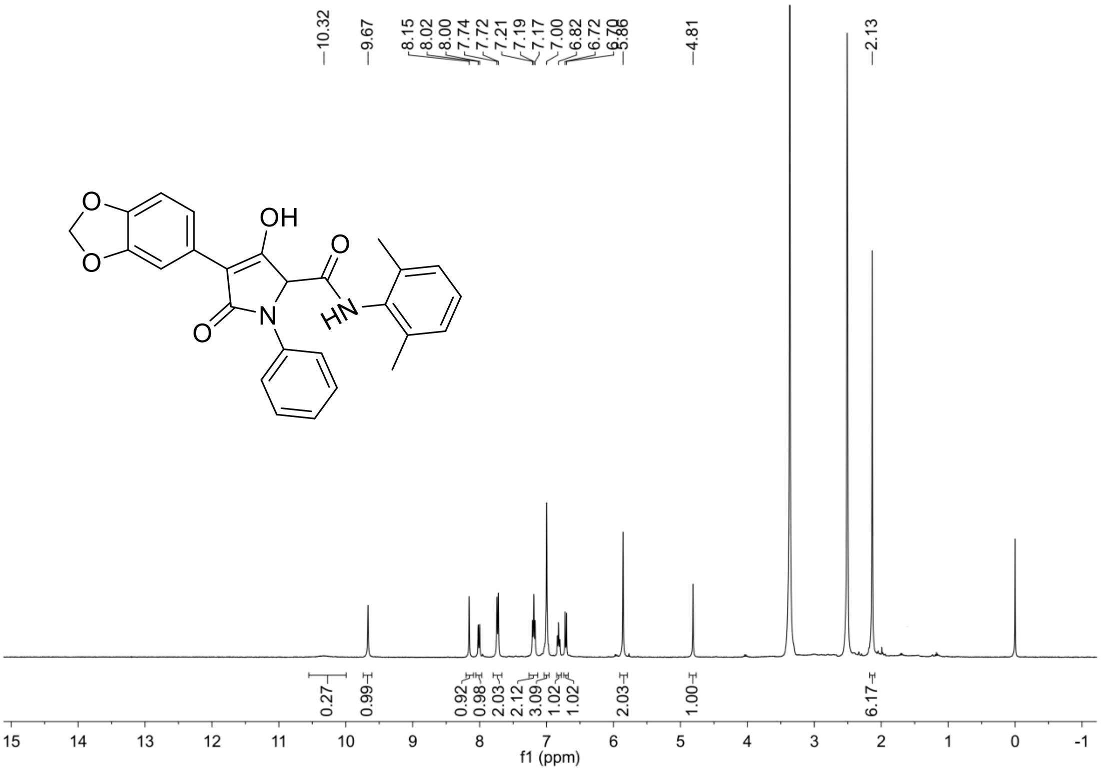

OH
O
O
O
O
N
HN
10.32
9.67
8.15
8.02
8.00
7.74
7.72
7.21
7.19
7.17
7.00
6.82
6.72
6.70
5.86
4.81
2.13
0.27
0.99
0.92
0.98
2.03
2.12
3.09
1.02
1.02
2.03
1.00
6.17
f1 (ppm)

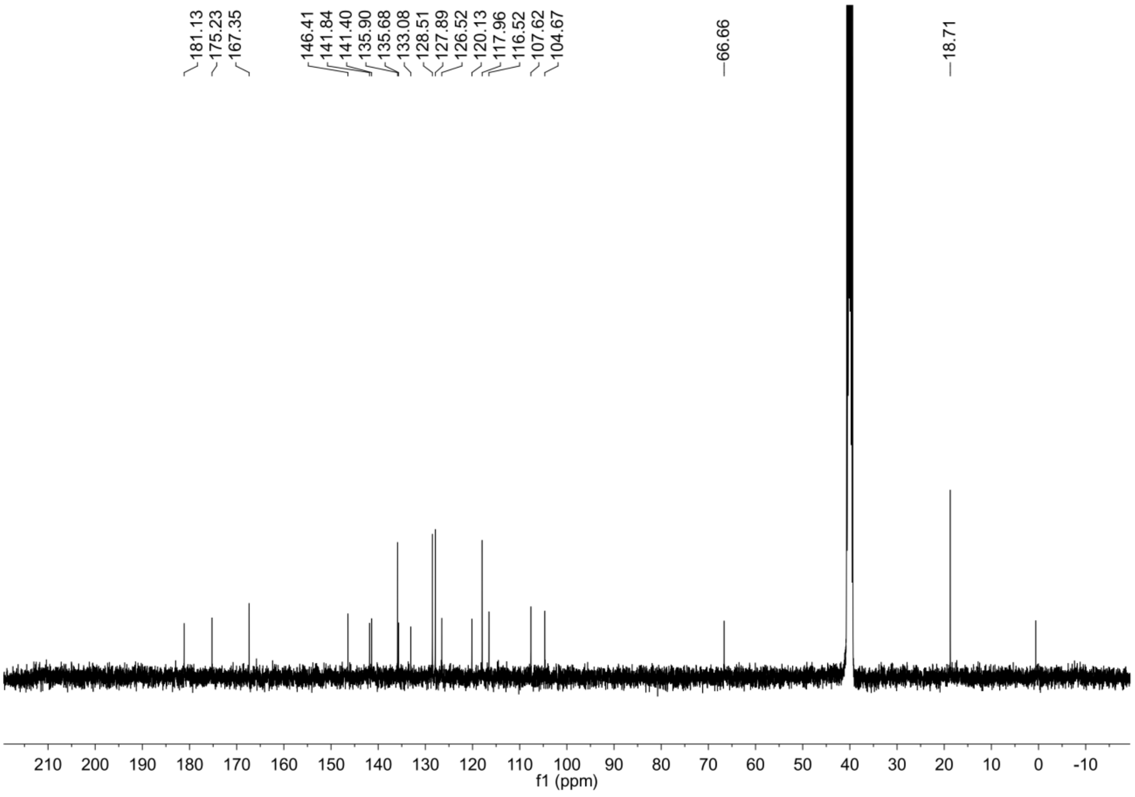

181.13
175.23
167.35
146.41
141.84
141.40
135.90
135.68
133.08
128.51
127.89
126.52
120.13
117.96
116.52
107.62
104.67
66.66
18.71
f1 (ppm)
